# Gas-Phase Construction of Compact Capping Layers for High-Performance Halide Perovskite X-Ray Detectors

**DOI:** 10.1007/s40820-025-01900-3

**Published:** 2025-12-20

**Authors:** Bin Zhang, Chuanyun Hao, Shoufeng Zhang, Bin Xue, Xiangfan Xie, Shengqiao Zeng, Bin Yang, Fang Xu, Hui Li, Xin’an Zhang, Zhang Qu, Kai-Hang Ye, Guangda Niu, Wallace C. H. Choy, Kezhou Fan, Kam Sing Wong, Lei Yan, Xingzhu Wang, Shuang Xiao, Cangtao Zhou

**Affiliations:** 1 Shenzhen Key Laboratory of Ultraintense Laser and Advanced Material Technology, Center for Intense Laser Application Technology (iLaT) and College of Engineering Physics, Shenzhen Technology University, Shenzhen, 518118 People’s Republic of China; 2 Engineering and Research Center for Integrated New Energy Photovoltaics & Energy Storage Systems of Hunan Province and School of Electrical Engineering, University of South China, Hengyang, 421001 People’s Republic of China; 3 School of Electrical Engineering, Guangxi University of Science and Technology, Liuzhou, 545006 People’s Republic of China; 4 Institute for Sustainable Transformation School of Chemical Engineering and Light Industry, Guangdong University of Technology, Guangzhou, 510006 People’s Republic of China; 5 Research Institute of Huazhong University of Science and Technology in Shenzhen, Shenzhen, 518052 People’s Republic of China; 6 Wuhan National Laboratory for Optoelectronics, Huazhong University of Science and Technology, Wuhan, 430074 People’s Republic of China; 7 Department of Electrical and Electronic Engineering, The University of Hong Kong, Pokfulam Road, Pokfulam, Hong Kong SAR People’s Republic of China; 8 Department of Physics and William Mong Institute of Nano Science and Technology, The Hong Kong University of Science and Technology, Clear Water Bay, Kowloon, Hong Kong SAR People’s Republic of China

**Keywords:** Halide perovskite, Ion migration, Interface, Heterostructure, X-ray detection

## Abstract

Halide perovskites have emerged as promising materials for X-ray detection with exceptional properties and reasonable costs. Among them, heterostructures between 3D perovskites and low-dimensional perovskites attract intensive studies of their advantages due to low-level ion migration and decent stability. However, there is still a lack of methods to precisely construct heterostructures and a fundamental understanding of their structure-dependent optoelectronic properties. Herein, a gas-phase method was developed to grow 2D perovskites directly on 3D perovskites with nanoscale accuracy. In addition, the larger steric hindrance of organic layers of 2D perovskites was proved to enable slower ion migration, which resulted in reduced trap states and better stability. Based on MAPbBr_3_ single crystals with the (PA)_2_PbBr_4_ capping layer, the X-ray detector achieved a sensitivity of 22,245 μC Gy_air_^−1^ cm^−2^, a response speed of 240 μs, and a dark current drift of 1.17 × 10^–4^ nA cm^−1^ s^−1^ V^−1^, which were among the highest reported for state-of-the-art perovskite-based X-ray detectors. This study presents a precise synthesis method to construct perovskite-based heterostructures. It also brings an in-depth understanding of the relationship between lattice structures and properties, which are beneficial for advancing high-performance and cost-effective X-ray detectors.

**Supplementary Information:**

The online version contains supplementary material available at 10.1007/s40820-025-01900-3.

## Highlights

- A gas-phase method has been developed, which can directly grow two-dimensional perovskite on three-dimensional perovskite with nanoscale precision.
- The steric hindrance of the organic layer within 2D perovskites influences the ion migration in lattice. Larger steric hindrance enables slower ion movement.
- The constructed 2D/3D heterojunction device showed ultra-high sensitivity, ultra-fast response speed, and ultra-low dark current.

**Supplementary Information:**

The online version contains supplementary material available at 10.1007/s40820-025-01900-3.

## Graphical abstract

**Figure:**
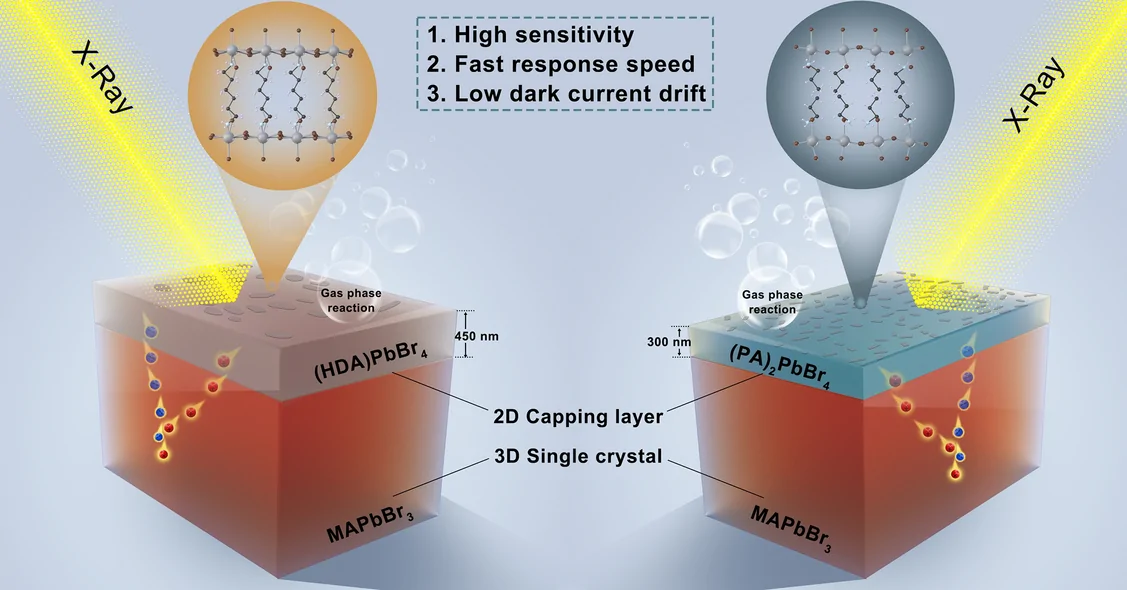

## Introduction

Medical X-ray imaging serves a critical function in accurate diagnosis and treatment of numerous medical conditions in humans [1–3]. However, there are risks associated with the use of X-ray. As a kind of ionizing radiation, X-ray has enough energy to potentially cause damage to DNA, as well as an increased possibility of cancer [4–6]. Such a possibility has a positive correlation to the total dose of X-ray exposure. Therefore, great efforts have been made to reduce the dose of medical X-ray imaging [6–8]. In the pursuit of this goal, X-ray detectors with outstanding performance are highly required with high sensitivity, fast response speed, low detection limit, and large signal-to-noise ratio (SNR) [1–4].

Halide perovskites have emerged as promising materials for X-ray detection with exceptional properties, such as strong X-ray absorption coefficient, long carrier lifetime, and high carrier mobility [7, 9]. Notably, the very low production cost of perovskite X-ray detectors further enhances their advantages, making them particularly attractive for a frequent replacement in case of massive usage such as radiation protection dosimeters [10]. In recent years, perovskite-based X-ray detectors have already demonstrated outstanding performances [1, 2, 11–13]. The α phase formamidinium lead iodide (α-FAPbI_3_) single-crystal (SC)-based X-ray detectors showed a high sensitivity of 4.15 × 10^5^ μC Gy_air_^−1^ cm^−2^ and a short response time of 214 μs (70–120 keV) [11]. The mixed-cation perovskite, Cs_0.05_FA_0.9_MA_0.05_PbI_3_, showed a high sensitivity of 1.5 × 10^4^ μC Gy_air_^−1^ cm^−2^ and a comparable short response time of 235 μs (59 keV) [2]. The mixed-cation perovskite, MA_0.42_FA_0.58_PbI_3_, showed an ultra-high sensitivity of 1.16 × 10^6^ μC Gy_air_^−1^ cm^−2^ and a relatively long response time of 2 × 10^7^ μs (22 keV) [12]. However, many halide perovskites suffer from severe ion migration, which leads to the instability of the baseline and response signals [13–15]. The reason is that halide perovskites are ionic crystals. Their constituent ions are bound to each other by relatively weak ionic bonds, which are relatively easy to move across the lattices. Besides, X-ray detectors are working at high electric fields, such as 80 and 200 V mm^−1^ [2, 11]. Such an electric field could drive the ions of halide perovskites moving at a notable speed and thus cause issues of stability. In another aspect, the surface of 3D perovskites is full of dangling bonds, a kind of deep-level surface defects [13, 16]. These defects not only facilitate recombination of X-ray-excited excess carriers (thereby reducing sensitivity), but moisture ingress represents a significant challenge to the long-term operational stability and performance fidelity of perovskite-based X-ray detectors [17]. Ambient water vapor readily permeates the perovskite lattice, initiating detrimental chemical reactions such as hydration and hydrolysis. This leads to irreversible degradation pathways including phase segregation, ion migration, and the formation of insulating PbI₂, ultimately manifesting as increased dark current, reduced charge carrier mobility, and diminished sensitivity over time. Crucially, the construction of 2D/3D heterojunctions effectively addresses this vulnerability. The hydrophobic organic spacers inherent in the 2D perovskite layer form a dense, highly crystalline barrier at the heterojunction interface. This barrier acts as a molecular sieve, significantly impeding the diffusion of water molecules toward the moisture-sensitive 3D perovskite active layer while maintaining efficient charge transport vertically. Consequently, this protective architecture substantially mitigates moisture-induced degradation, enhancing device robustness and operational lifetime. Under high electric fields, these defects further induce undesirable dark current, posing additional hurdles for practical X-ray detector applications [18].

To overcome the above-mentioned problems, the integration of 3D perovskites with low-dimensional perovskites has been studied, as it effectively suppresses ion migration, thereby enhancing device stability and performance [13, 19]. Previously, we developed a gradient 2D-3D layered perovskite films for X-ray detection [20]. The ion migration was largely suppressed, and the defect density was also reduced with the 2D–3D layered structure, while the sensitivity showed no increase. In another work, 4-fluorophenethylammonium lead bromide-formamidinium lead bromide (FPEA_2_PbBr_4_-FAPbBr_3_)-based X-ray detectors also showed suppressed ion migration, reduced defect density, and comparable sensitivity, compared with FAPbBr_3_-based X-ray detectors [21]. The 3D/2D structure could significantly suppress ion migration and reduce defect density, while the sensitivity was not increased to a satisfactory level. We noted that the thickness of 2D perovskites in these works was normally several micrometers or larger. Considering that 2D perovskite also showed two orders of magnitude lower sensitivity than their 3D counterparts, their thickness should be reduced to achieve the effects of ion migration suppression and carrier transport improvement simultaneously for optimal detection performance [13]. This limitation stems from the inherit properties of 2D perovskites: Strong quantum confinement and large exciton binding energies drastically reduce carrier mobility. Consequently, when the 2D capping layer thickness substantially exceeds the carrier diffusion length, severe charge collection losses occur, leading to significantly compromised sensitivity. Implementing an ultrathin, nanoscale 2D capping layer offers a viable strategy to mitigate these transport limitations while simultaneously passivating surface dangling bonds on the 3D perovskite, suppressing deep-level defects, and minimizing non-radiative recombination. Compared with electrons and holes, ions have significantly larger radius, which can be effectively restrained by large-sized amine layers [22]. In this regard, a thin and compact 2D layer in heterostructures should effectively inhibit ion migration. Besides, the construction of thin and high-quality 2D perovskites layer directly on 3D perovskites is still challenging. Therefore, it is highly required to develop strategies to enable controllable growth of 2D perovskite layer in nanoscale directly on 3D perovskites.

In addition, there is still a lack of in-depth understanding of the relationship between properties and structures of 2D perovskites in 3D/2D heterojunctions. Current researches about perovskite-based X-ray detectors are scattered to study limited species of 2D perovskites [20, 21, 23]. We have known little about the influence of lattice spacing, distortion of organic molecules, and electric dipoles toward ion migration, carrier transport, and formation of defects in 3D/2D heterojunctions. As a result, it is hard to conceive a clear methodology to design 2D perovskites with optimal properties for X-ray detection. Thus, plenty of works are in great demand to unveil the above-discussed structure–property relationships.

Herein, we demonstrate a gas-based method to synthesize fully covered and pinhole-free 2D perovskite capping layers directly on a 3D perovskite with thickness in nanoscale, including propylammonium lead bromide ((PA)_2_PbBr_4_) and 1,6-hexanediammonium lead bromide ((HDA)PbBr_4_). This method should be generalizable to various 3D perovskites, such as MAPbI_3_ and FAPbI_3_. Methylammonium lead bromide (MAPbBr_3_) was specifically selected as the 3D layer due to its better stability compared to other perovskites. Besides, the preparation process of MAPbBr_3_ is relatively straightforward and can be achieved at lower temperatures, minimizing fabrication complexity and energy consumption. The as-synthesized 2D perovskites are pure *n* = 1 phase with different growth rates depending on the size of A-site molecules. With the 2D perovskite capping layers, the defect states of the 3D perovskite were largely reduced and a preferred staggered gap was formed at the interface. Thus, we could achieve a sensitivity of 22,245 μC Gy_air_^−1^ cm^−2^ and a response speed of 240 μs with the (PA)_2_PbBr_4_ capping layer, while MAPbBr_3_-based X-ray detectors without capping layers only showed a sensitivity of 2,137 μC Gy_air_^−1^ cm^−2^ and a response speed of 421 μs. Besides, the nanoscale-thin 2D perovskite capping layers also showed a remarkable ion migration suppression effect. The dark current drift of (PA)_2_PbBr_4_-MAPbBr_3_-based detectors was significantly reduced to 1.17 × 10^–4^ nA cm^−1^ s^−1^ V^−1^. Moreover, detectors with the (PA)_2_PbBr_4_ capping layer showed better performance than detectors with the (HDA)PbBr_4_ capping layer, which was caused by the structural superiority of closely stacked PA ions.

## Experimental Section

### Materials

Lead bromide (PbBr_2_, > 98%) was purchased from Alfa Aesar. N,N-dimethylformamide (DMF, > 99.5%) and isopropyl alcohol (IPA, ≥ 99.7%) were purchased from Greagent. Methylammonium bromide (MABr, ≥ 99%) was purchased from Great Cell Solar. Propylamine (PA, ≥ 99%) and 1,6-hexanediamine (HDA, ≥ 99%) were purchased from Adamas. C_60_ was purchased from Xi’an Polymer Light Technology. All chemicals were used as received without further purification.

### Preparation of MAPbBr_3_ Single Crystal, (PA)_2_PbBr_4_ and (HDA)PbBr_4_ Capping Layers

#### ***Preparation of MAPbBr***_***3***_*** Single Crystal***

1.388 g (0.0125 mol) of MABr and 4.590 g (0.0125 mol) of PbBr_2_ and 10 mL of DMF were mixed in a 40-mL beaker. Then, the beaker was sealed with parafilm and aluminum foils. The mixed solution was stirred on a magnetic stirrer at 500 r min^−1^ for 4 h. The well-mixed solution was filtered with a PTFE filter (0.45 μm) and then added 100 µL IPA. To obtain seed crystals, the solution was directly placed on a hot plate at 85 °C for 30 min. The as-prepared seed crystals were collected and dried for storage. For the single-crystal growth, one seed crystal was added into the as-prepared precursor solution. Then, the precursor solution with seed crystals was heated to 44 °C in an oil bath. During the oil bath, the temperature needs to be maintained for 24 h and then should be increased by 1 °C every 24 h [24].

#### ***Preparation of (PA)***_***2***_***PbBr***_***4***_*** and (HDA)PbBr***_***4***_*** Capping Layers***

A single crystal with dimensions of approximately 10 mm × 10 mm × 2 mm was cut into two pieces using a cutting machine with a 0.25 mm diamond wire. The surface of the single crystals was manually polished using 7000-grit sandpapers. Then, the polished crystals were annealed at 100 °C for 1 h in nitrogen. For (PA)_2_PbBr_4_ capping layer synthesis, 50 μL of PA was added into a glass bottle (100 mL) and fully evaporated at room temperature. Then, single crystals were covered by the bottles with PA vapor to enable the reaction between PA and MAPbBr_3_. The thickness of (PA)_2_PbBr_4_ capping layers could be tuned by adjusting reaction time, and the optimal time for PA was 40 s. For (HDA)PbBr_4_ capping layer synthesis, 50 μL of HDA was added into a glass bottle (100 mL) and fully evaporated in an oven at 60 °C. Then, single crystals were covered by the bottles with HDA vapor to enable the reaction between HDA and MAPbBr_3_. The thickness of (HDA)PbBr_4_ capping layers could be also tuned by adjusting reaction time, and the optimal time for HDA was 180 s.

### Device Fabrication

To fabricate an X-ray detector, a 100-nm-thick Au electrode was thermally evaporated on the samples in a vacuum chamber with pressure less than 4 × 10^–4^ Pa. The exposure area to X-ray was 0.01365 cm^2^, and the area of gold electrode was 0.0085 cm^2^. The as-fabricated detector is shown in Fig. S1. For fabricating space charge limited current (SCLC) devices, a layer of 10 nm thick C_60_ was thermally evaporated on the samples surface, and then, a 100-nm-thick Ag electrode was thermally evaporated on the layer of C_60_ in a vacuum chamber with pressure less than 4 × 10^–4^ Pa.

### Material Characterizations

The X-ray diffraction (XRD) patterns are obtained using a Rigaku SmartLab X-ray diffractometer with Cu-Kα radiation. Scanning is performed from 5° to 80° at 45 kV and 200 mA with a scanning rate of 10 ° min^-1^. Scanning electron microscope (SEM) images were obtained via a field-emission SEM (Zeiss, Gemini SEM 300). Both of the planar samples and cross-sectional samples were tested under identical conditions: electron high tension of 5.00 kV, InLens signal detection mode, and system vacuum below 1 × 10⁻^6^ mbar. The EDS line scanning analysis was acquired along selected paths spanning 600 nm using an accelerating voltage of 15 kV. Atomic force microscope (AFM) measurements were conducted via Bruker Dimension Icon and measured with OMCL-AC160TS-R3 type tips (Spring constant: 26 N m^−1^). The time-resolved photoluminescence (TRPL) spectra were determined with a time-correlated single-photon counting system (Edinburgh Instruments, FLS1000), using pulsed laser (375 nm) as the excitation source with a pulse width of 60 ps and a pulse repetition frequency of 0.5 MHz. UV–vis absorption was performed using Thermo Fisher's Evolution 220, with a scanning wavelength range of 750 to 400 nm. The steady-state photoluminescence (PL) test was monitored by F-2700 (Hitachi, Japan). The wavelength of excitation light was 360 nm. The start wavelength of emission spectra was 380 nm, and the end wavelength is 600 nm with a scanning speed of 1,500 nm min^−1^. The excitation slit was 20 nm, and the emission slit was 2.5 nm. The ultraviolet photoemission spectra (UPS) measurements were conducted via ESCALAB Xi + (Thermo Fisher Scientific). The helium Iα radiation was used for UPS characterization, and the photon energy was 21.22 eV. The instrument was performed under Constant Analyzer Energy–Pass Energy 1.0 eV analyzer mode with 0.020 eV energy step size.

### UPS Data Analysis

The cutoff edge manifests as an inflection point with a sharp decrease in intensity, which determined the cutoff energy (*E*_cutoff_). The Fermi edge manifests as a boundary where the intensity abruptly transitions from nonzero to zero. The Fermi edge energy (*E*_Fermi_) was determined by the position of the intersection of the tangent of the edge and the baseline.

The Fermi level (*E*_F_) can be calculated by the following equation:

1 $$E_{{\mathrm{F}}} = E_{{{\mathrm{photon}}}} {-}E_{{{\mathrm{cutoff}}}}$$

where *E*_photon_ is the photon energy of the helium Iα radiation (21.22 eV).

The valence band maximum (VBM, *E*_V_) can be calculated by the following equation:

2 $$E_{{\mathrm{V}}} = E_{{\mathrm{F}}} + E_{{{\mathrm{Fermi}}}}$$

The conduction band minimum (CBM, *E*_C_) can be calculated by the following equation:

3 $$E_{{\mathrm{C}}} = E_{{\mathrm{V}}} {-}E_{{\mathrm{g}}}$$

where *E*_g_ is the bandgap.

### Trap Density Characterization

SCLC was carried out on electron-only devices (Ag/C_60_/perovskite/C_60_/Ag). Keithley 2400 was used to collected the current–voltage curves in dark. Specifically, in the linear ohmic region of the dark current–voltage curve, the average electron trap density was obtained from the following formula:

4 $${n}_{\mathrm{trap}}=\frac{2{V}_{\mathrm{TFL}}\varepsilon {\varepsilon }_{0}}{e{L}^{2}}$$

where *ε* is 25.5 for MAPbBr_3_ [25], *ε*_0_ is vacuum permittivity, *e* is the elementary charge, *L* is the length of gap, and *V*_TFL_ is the voltage of the trap-filled limit.

### Measurement of the Ion Migration Activation Energy

To assess the ion migration level in this work, temperature-dependent conductivity was measured in dark. Generally, electronic conduction is the main cause of conductivity at low temperature as the ion migration is suppressed. In this work, the ion migration rate was characterized by the activation energy (*E*_a_) of ionic conductivity, calculated using the Nernst–Einstein equation from the temperature-dependent measurements:

5 $$\sigma T = \frac{{\sigma_{0} }}{T}{\mathrm{exp}}\left( {\frac{{ - E_{{\mathrm{a}}} }}{{k_{B} T}}} \right)$$

where *k*_B_ is the Boltzmann constant, *σ*_0_ is a constant, and *T* is the temperature. The experimental setup used in this work consisted of a Tektronix 2450 source meter and a Lakeshore cryoprobe station with a Lakeshore 336 temperature controller. The conductivities are recorded in the temperature range of 120 to 300 K, in 10 K steps. The conductivities of the devices were extracted by measuring the *I*-*t* curve at 0.5 V.

### Measurement of X-ray Detector Performance

X-ray detection performance was measured in a dark box made of lead to minimize electromagnetic and ambient light disturbance. A tungsten anode X-ray tube (Varex 160, VAREX) was used as the X-ray source. The X-ray tube was operated under a fixed 60 kV voltage, and the current was adjusted from 6.4 to 0.1 mA to change the X-ray dose rate. Several 0.5-mm-thick steel sheets were used as the attenuator to generate low X-ray dose rates. The X-ray dose rate was carefully calibrated using the AT1123 radiation dosimeter manufactured by ATOMTEX (Belarus). During the performance measurements, the X-ray response current was collected using a precision source meter (Keithley 2400). During the measurement of response time, the OE1B10 mechanical chopper (Guangzhou SSI instruments) with a frequency of 1000 Hz for X-ray chopping was activated (Fig. S2). The response rate of perovskite under pulse bias with a bandwidth of 0.5 s and a voltage of 10 V was recorded in waveform-meas mode of the 4200 source meter (Keithley, Tektronix).

All of the above measurements of the X-ray detectors are performed in the ambient atmosphere environment (temperature ~ 25 °C and relative humidity ~ 40 RH%), and none of the detectors in this work were encapsulated.

The SNR was calculated with the following equation [13]:

6 $${\mathrm{SNR}}= \frac{{I}_{s}}{{I}_{n}}$$

where *I*_*s*_ represents the disparity between the average current under X-ray and the average dark current, while *I*_*n*_ denotes the noise current. The latter is quantified as the standard deviation of the X-ray response current, which can be mathematically expressed as:

7 $${I}_{n}=\sqrt{\frac{1}{N}\sum_{i}^{N}{({I}_{i}-{I}_{d})}^{2}}$$

where *I*_i_ represents the measured dark current *I*_d_ represents the average dark current.

## Results and Discussion

### Suppressed Formation of Dangling Bonds by 2D Perovskites

**Fig. 1:**
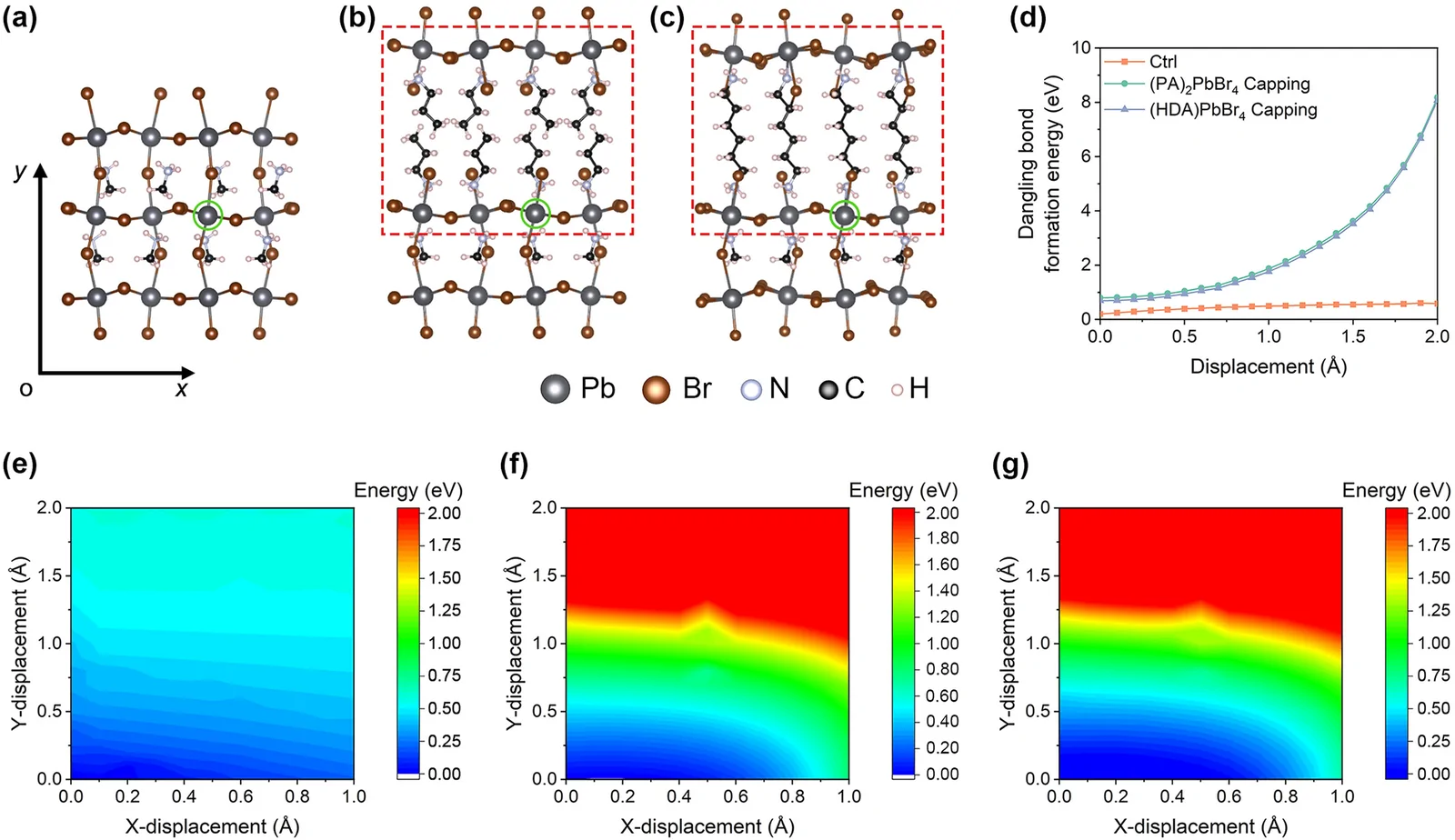
Suppressed formation of dangling bonds by 2D perovskites. Calculated models for dangling bonds formation simulation: **a** MAPbBr_3_, **b** (PA)_2_PbBr_4_ on top of MAPbBr_3_, **c** (HDA)PbBr_4_ on top of MAPbBr_3_. The axis plane, defined by the *x* and *y* axes, indicates the migration plane of Br^−^. The *x* axis corresponds to the crystal axis along *a*-direction. The *y* axis corresponds to the crystal *c*-axis oriented along the out-of-plane direction. **d** Dangling bond formation energy curves versus the displacement of Br^−^ along Y-axis with different structures. The displacement of Br^−^ along X-axis is 1 Å. “Ctrl” refers to MAPbBr_3_. “(PA)_2_PbBr_4_ capping” refers to (PA)_2_PbBr_4_ on top of MAPbBr_3_. “(HDA)PbBr_4_ capping” refers to (HDA)PbBr_4_ on top of MAPbBr_3_. Mapping of dangling bond formation energy with the displacement of Br^−^ in the *x*-o-*y* plane: **e** MAPbBr_3_, **f** (PA)_2_PbBr_4_ on top of MAPbBr_3_, **g** (HDA)PbBr_4_ on top of MAPbBr_3_

The conventional MAPbBr_3_ consists of MA^+^, Pb^2+^, and Br^−^ ions with a 3D perovskite-type lattice structure (Fig. 1a). When replacing the MA^+^ with large amine cations, the 3D lattice could transform into a 2D lattice with a layered structure [9, 26]. Herein, the candidates for the large A-site cations of 2D perovskites are PA and HDA (Scheme S1). The PA^+^ could form a Ruddlesden–Popper (RP) phase perovskite with Pb^2+^ and Br^−^ ions, which has a formula of (PA)_2_PbBr_4_. In the (PA)_2_PbBr_4_ lattice, there are two layers of PA^+^ cations between the [PbBr_4_]^2−^ sheets (Fig. 1b, red dashed rectangle) [9, 26]. The HDA^2+^ could form the Dion–Jacobson (DJ) phase perovskite with Pb^2+^ and Br^−^ ions, which has a formula of (HDA)PbBr_4_. In the (HDA)PbBr_4_ lattice, there is only one layer of HDA^2+^ cations between the [PbBr_4_]^2−^ sheets (Fig. 1c, red dashed rectangle) [9, 26]. In this way, we could obtain two kinds of 2D perovskites with the same number of carbon and nitrogen atoms between the [PbBr_4_]^2−^ sheets, while the steric hindrance of organic layers of (PA)_2_PbBr_4_ is larger than that of (HDA)PbBr_4_, as PA^**+**^ cations have a higher crystal space utilization rate than HDA^2+^ cations.

To investigate the influence of the 2D perovskites on MAPbBr_3_, we conducted a simulation based on three configurations: 1) conventional MAPbBr_3_, 2) (PA)_2_PbBr_4_ layers on top of MAPbBr_3_, and 3) (HDA)PbBr_4_ layers on top of MAPbBr_3_ (Fig. 1a–c). The formation energy of dangling bonds of Pb^2+^ ions could be an indicator of this influence. Thereafter, we designed a model in which moving Br^−^ ions away from the Pb^2+^ ions, highlighted by green circles, allowed us to calculate the dangling bond formation energy versus the displacement of Br^−^ in two directions (Fig. 1a–d). When the displacement of the Br^−^ ion in Y-axis increases, the dangling bond formation energy of MAPbBr_3_ increases with a much slower rate compared with MAPbBr_3_ with 2D layers (Fig. 1d). Besides, the dangling bond formation energy of MAPbBr_3_ with (PA)_2_PbBr_4_ layers is slightly higher than that of MAPbBr_3_ with (HDA)PbBr_4_ layers. The larger the dangling bond formation energy is, the harder it is for dangling bonds to form. In other words, MAPbBr_3_ with (PA)_2_PbBr_4_ layers or (HDA)PbBr_4_ layers should have fewer defects than conventional MAPbBr_3_ at the interface.

The dangling bond formation energy with Br^−^ ions moving in the *x*-*o*-*y* plane was also investigated (Fig. 1a–c, the axis indicate the *x*-*o*-*y* plane). In the MAPbBr_3_ lattice, the dangling bond formation energy could hardly exceed 0.5 eV in most areas, indicating that the Br^−^ ions are relatively easy to migrate (Fig. 1a, e). In stark contrast, the dangling bond formation energy in the MAPbBr_3_-(PA)_2_PbBr_4_ lattice could quickly exceed 0.5 eV with ~ 0.5 Å displacement along the Y-axis, indicating the remarkable influence of 2D layers toward defects formation. In the MAPbBr_3_-(PA)_2_PbBr_4_ lattice, the dangling bond formation energy along the X-axis increases at a slower rate than that along the Y-axis, indicating the Br^−^ ions are easier to move along the X-axis compared to its movement along the Y-axis. In all, the blue area (the dangling bond formation energy ≤ 0.5 eV) in Fig. 1f for MAPbBr_3_-(PA)_2_PbBr_4_ is much smaller than that in Fig. 1e for MAPbBr_3_, which indicates that the Br^−^ ions are tightly constrained to the Pb^2+^ ion. In this way, the ion migration speed of Br^−^ ions in the MAPbBr_3_-(PA)_2_PbBr_4_ lattice should be much slower than that in the conventional MAPbBr_3_ lattice.

For the configuration of (HDA)PbBr_4_ layers on top of MAPbBr_3_, its dangling bond formation energy shows a quick increase along the Y-axis and a relatively slow increase along the X-axis, which is similar to the situation for MAPbBr_3_-(PA)_2_PbBr_4_. Besides, the blue area in Fig. 1g for MAPbBr_3_-(HDA)PbBr_4_ is smaller than that in Fig. 1f for MAPbBr_3_-(PA)_2_PbBr_4_, which implies that the Br^−^ ions in the MAPbBr_3_-(HDA)PbBr_4_ lattice are relatively easier to migrate compared to the Br^−^ ions in the MAPbBr_3_-(PA)_2_PbBr_4_ lattice. The origin of this difference should be the larger steric hindrance of the organic layer of (PA)_2_PbBr_4_ lattice than that of the (HDA)PbBr_4_ lattice (Fig. 1b, c). Combined with the high crystal space utilization rate of PA⁺ and the high dangling bond formation energy, it can be inferred that the amino group (-NH₃⁺) of PA⁺ exhibits stronger coordination ability with Pb^2^⁺, forming more stable interfacial bonding and higher Coulombic forces to directly bind Br⁻ ions [26]. In contrast, the bis-amino structure of HDA^2^⁺ may lead to charge delocalization, resulting in weaker interactions with Pb^2^⁺ and less effective interfacial electrostatic confinement compared to PA⁺ [27, 28]. Nevertheless, the ion migration speed of Br^−^ ions in the MAPbBr_3_-(HDA)PbBr_4_ lattice should be still much slower than that in the conventional MAPbBr_3_ lattice (Fig. 1e, g). Based on the simulation results, the defect formation and ion migration should be largely suppressed of MAPbBr_3_ with the (PA)_2_PbBr_4_ and (HDA)PbBr_4_ layers. Besides, the MAPbBr_3_ with the (PA)_2_PbBr_4_ should be the best among the three configurations considered in this research.

### Morphology and Crystal Structures

Our previous research demonstrated that formamidine (FA) vapor could evenly react with the 400-nm-thick perovskite film and uniformly diffuse into its lattice [29, 30]. Besides, the migration speed of cations has a reverse relationship to their sizes [9, 31]. Applying this relationship, PA and HDA should exhibit a lower migration speed inside the perovskite lattice than FA does. Thus, the depth of replacement reaction between amines and perovskites should be finely controlled by the size-dependent diffusion speed.

**Fig. 2:**
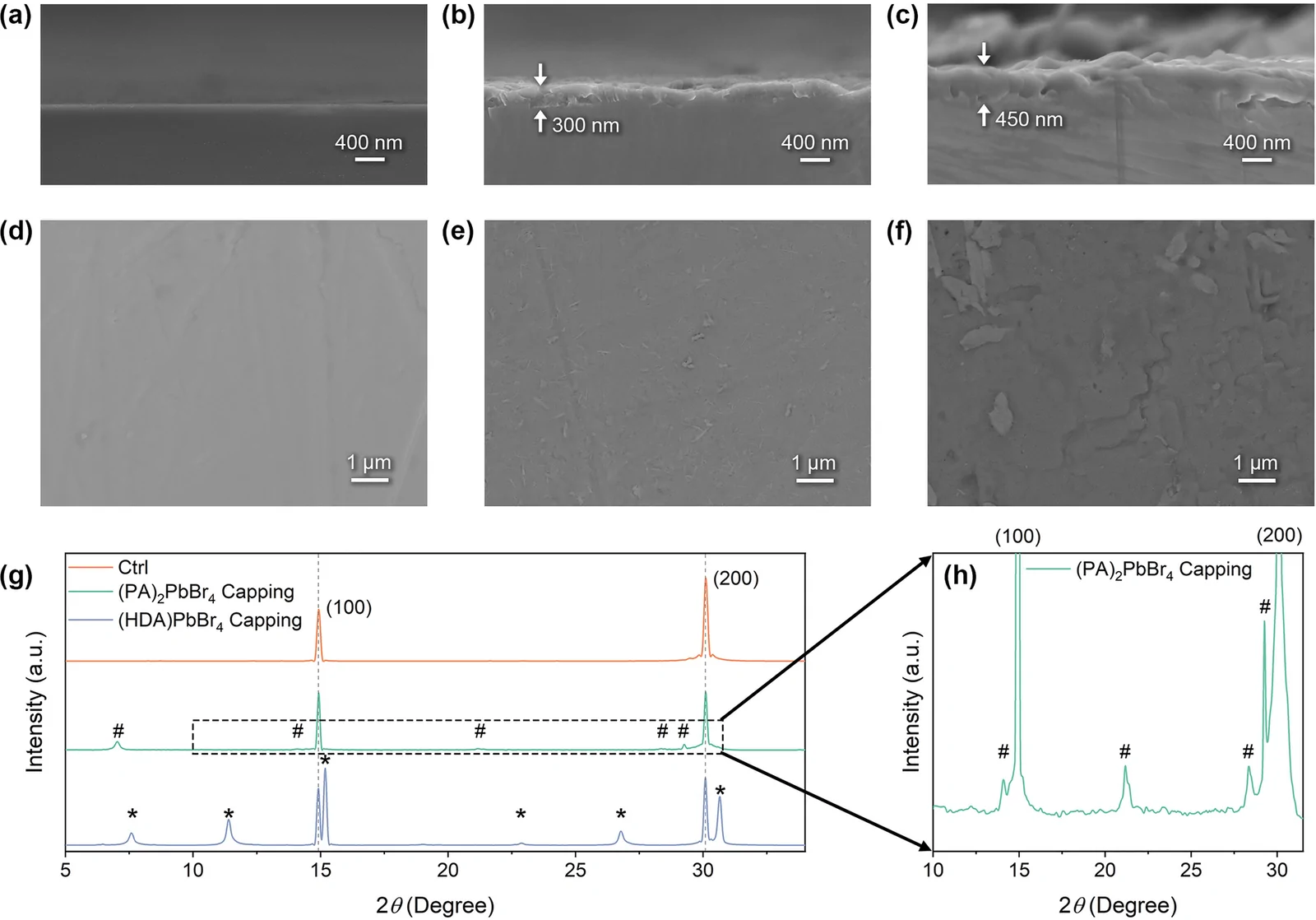
Morphology and crystal structures. Side-view scanning electron microscope (SEM) images of **a** MAPbBr_3_ single crystal (Ctrl sample), **b** MAPbBr_3_ single crystal with the (PA)_2_PbBr_4_ capping layer ((PA)_2_PbBr_4_ capping sample), and **c** MAPbBr_3_ single crystal with the (HDA)PbBr_4_ capping layer ((HDA)PbBr_4_ capping sample). The scale bar is 400 nm. Top-view SEM images of **d** Ctrl, **e** (PA)_2_PbBr_4_ capping, and **f** (HDA)PbBr_4_ capping samples. The scale bar is 1 μm. **g** X-ray diffraction (XRD) patterns of Ctrl, (PA)_2_PbBr_4_ capping, and (HDA)PbBr_4_ capping samples. **h** Enlarged XRD pattern of (PA)_2_PbBr_4_ capping sample to clearly show the peaks of (PA)_2_PbBr_4_. Hash signs: XRD peaks of (PA)_2_PbBr_4_. Asterisk signs: XRD peaks of (HDA)PbBr_4_

The optimized reaction time between PA and MAPbBr_3_ is 40 s. After the reaction, a ~ 300-nm-thick capping layer appeared on top of the MAPbBr_3_ single crystal (Fig. 2a, b). The optimized reaction time between HDA and MAPbBr_3_ is 180 s, which is over 3 times larger than the optimal reaction time of PA. However, the thickness of the HDA capping layer is ~ 450 nm, only 50% larger than the thickness of the PA capping layer (Fig. 2c). Assuming the reaction speed is nearly constant during the whole process, the growth rate of the capping layer was ~ 7.5 and ~ 2.5 nm s^−1^ for PA and HDA, respectively. Considering that the size of PA molecule is about half of the size of the HDA molecule, the growth rate of the capping layer had a negative correlation to the size of amines, which was in good agreement with our hypothesis mentioned in the above part (Scheme S1). By varying the reaction time, 2D perovskite layers of different thicknesses were synthesized on the MAPbBr_3_ SCs (Figs. 2b, c and S3). Then, we systematically determined the optimal reaction time by analyzing the sensitivity of devices incorporating 2D perovskite layers with varying thicknesses (Fig. S4). The linear scanning of heterostructures in cross section was performed by employing energy-dispersive spectrometer (EDS). In Fig. S5a, the (PA)_2_PbBr_4_ capping sample shows a gradually decreased Br/Pb molar ratio from 3.38 to 2.24, corresponding to 2D perovskite layer (theoretical Br/Pb molar ratio: 4) and 3D perovskite layer (theoretical Br/Pb molar ratio: 3). In Fig. S5b, the (HDA)PbBr_4_ capping sample also showed a similar gradually decreased Br/Pb molar ratio, confirming the formation of the 2D/3D heterojunction.

Apart from the thickness, the morphology of capping layers was also dependent on the species of amines. The fine-polished MAPbBr_3_ single crystal showed a smooth surface with several scratches made by sandpaper (Figs. 2d and S6a). After the PA replacement reaction, a smooth capping layer formed with several nanoscale flakes (Fig. 2e). The scratches could also be observed, indicating the conformal growth of the capping layer. When reacted with HDA, the surface showed rectangular grains in the microscale, which was significantly different to the morphology of PA capping layers (Fig. 2f). Nevertheless, both of PA capping and HDA capping layers fully covered the MAPbBr_3_ surface without any pinholes or cracks, which can be also confirmed by the AFM images (Fig. S6).

The MAPbBr_3_ single crystal exhibited a pure phase XRD pattern with 2*θ* peaks at 14.94° and 30.11°, which corresponded to the (100) and (200) planes, respectively (Fig. 2g, orange line). For the MAPbBr_3_ single crystal with PA capping layer, 5 additional peaks appeared at 7.04°, 14.10°, 21.14°, 28.32°, and 29.28°, which were the diffraction peaks of (PA)_2_PbBr_4_ (Fig. 2g, h, teal green line, hash sign) [32]. The spacing of lattice planes along its *c*-axis was 25.09 Å, which could be calculated from the 2*θ* value (7.04°) of its (002) plane [33]. For the MAPbBr_3_ single crystal with HDA capping layer, 6 additional peaks appeared at 7.60°, 11.40°, 15.20°, 22.90°, 26.78°, and 30.68°, which were the diffraction peaks of (HDA)PbBr_4_ (Fig. 2g, blue-gray line, asterisk sign). The spacing of lattice planes along its *c*-axis was 23.25 Å, which could be also calculated from the 2*θ* value (7.60°) of its (002) plane. The 2D halide perovskites can be regarded as superlattices of quantum wells, in which the organic layers act as a barrier to out-of-plane electron motion [34]. Considering the similar spacing of lattice planes along its* c*-axis, the carrier transport of (PA)_2_PbBr_4_ and (HDA)PbBr_4_ is expected to be similar. Additionally, all the XRD patterns showed strong diffraction peaks of MAPbBr_3_. The reason for this phenomenon is the capping layer is thin, which is nearly “transparent” to X-ray. For simplicity, the pure MAPbBr_3_ single crystal is named the “Ctrl” sample. The MAPbBr_3_ single crystal with (PA)_2_PbBr_4_ capping layers is named the “(PA)_2_PbBr_4_ capping” sample. The MAPbBr_3_ single crystal with (HDA)PbBr_4_ capping layers is named the “(HDA)PbBr_4_ capping” sample.

### Optical and Electronic Properties

**Fig. 3:**
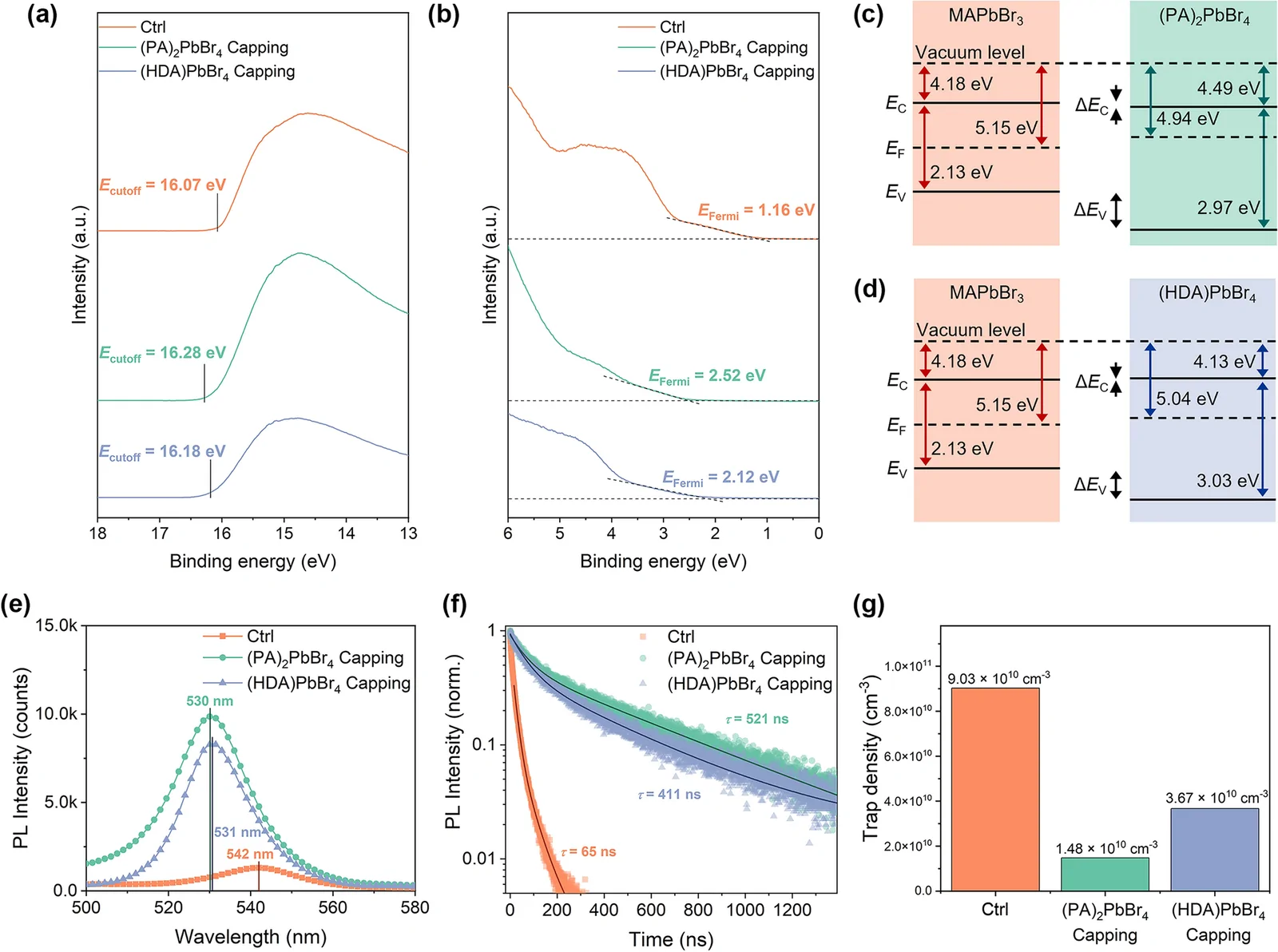
Optical and electronic properties. Ultraviolet photoelectron spectroscopy (UPS) spectra of Ctrl, (PA)_2_PbBr_4_ capping, and (HDA)PbBr_4_ capping samples: **a** cutoff edges, **b** Fermi edges. Schematic band alignment of **c** MAPbBr_3_-(PA)_2_PbBr_4_ heterojunction, **d** MAPbBr_3_-(HDA)PbBr_4_ heterojunction. **e** Steady-state photoluminescence (PL) spectra of Ctrl, (PA)_2_PbBr_4_ capping, and (HDA)PbBr_4_ capping samples. **f** Time-resolved photoluminescence (TRPL) spectra of Ctrl, (PA)_2_PbBr_4_ capping, and (HDA)PbBr_4_ capping samples. **g** Trap density of Ctrl, (PA)_2_PbBr_4_ capping, and (HDA)PbBr_4_ capping samples, which were calculated based on the space charge limited current (SCLC) method

Band alignment plays an important role in the carrier transport process of heterojunctions [9]. The cutoff energy of UPS of Ctrl, (PA)_2_PbBr_4_ capping, and (HDA)PbBr_4_ capping samples was 16.07, 16.28, and 16.18 eV, respectively (Fig. 3a). Thus, the Fermi levels of the three samples were calculated to be 5.15, 4.94, and 5.04 eV, respectively. Then, the valence band maximum (VBM) could be calculated from the Fermi energy and its difference to VBM (Fig. 3b). The VBM of Ctrl, (PA)_2_PbBr_4_ capping, and (HDA)PbBr_4_ capping samples was 6.31, 7.46, and 7.16 eV, respectively. The ultraviolet visible absorbance spectra of all samples only showed the optical properties of MAPbBr_3_ (Fig. S7). Thus, we could only obtain the optical bandgap of MAPbBr_3_ (Fig. S7a). To draw the band alignment scheme of (PA)_2_PbBr_4_ capping and (HDA)PbBr_4_ capping samples, we used both of our experimental results and reported data (Figs. S7 and S8) [34–40].

The Fermi level of the Ctrl sample was 0.97 eV lower than its conduction band minimum (CBM) and was 1.16 eV higher than its VBM, which indicates the MAPbBr_3_ single crystal is weak n-type semiconductor (Fig. 3c). The Fermi level of (PA)_2_PbBr_4_ was 0.45 eV lower than its CBM and 2.52 eV higher than its VBM, which indicates it was also a n-type semiconductor. When the heterojunction forms, electrons will transfer from (PA)_2_PbBr_4_ (with a lower work function, where the Fermi level is relatively closer to the vacuum level) to MAPbBr_3_ (with a higher work function, where the Fermi level is relatively farther from the vacuum level) at the interface to align their Fermi levels. In this way, (PA)_2_PbBr_4_ and MAPbBr_3_ could form a staggered gap (type II) (Fig. S9) [9]. The aligned energy band diagrams with a flat fermi level as the reference show the band bending at interface, which can facilitate the separation of electrons and holes, improving the device's performance in terms of carrier collection (Fig. S9). The band alignment of the (HDA)PbBr_4_-MAPbBr_3_ junction was also staggered, while the energy drop at its interface was smaller than that of the (PA)_2_PbBr_4_-MAPbBr_3_ junction (Figs. 3d and S9). A smaller energy drop normally indicates a weaker built-in electrical field and thus slower charge separation [9, 41]. In this way, the (PA)_2_PbBr_4_-MAPbBr_3_ junction is expected to show better charge separation efficiency than the (HDA)PbBr_4_-MAPbBr_3_ junction.

For the Ctrl sample, PL peak of MAPbBr_3_ is located at 542 nm with an intensity of ~ 1300 counts (Fig. 3e, orange line). With the (PA)_2_PbBr_4_ capping layer, the PL peak location of MAPbBr_3_ shifted to 530 nm and the intensity of the PL peak increased to ~ 10,000 counts (Fig. 3e, teal green line). With the (HDA)PbBr_4_ capping layer, the PL peak location of MAPbBr_3_ shifted to 531 nm and the intensity of the PL peak increased to ~ 8200 counts (Fig. 3e, blue-gray line). The blue-shifted PL peak and increased peak intensity indicated that the trap states of MAPbBr_3_ were largely reduced [42–45]. Besides, TRPL spectra showed that the PL lifetime of MAPbBr_3_ remarkably increased from 65 to 521 ns and 411 ns for (PA)_2_PbBr_4_ capping and (HDA)PbBr_4_ capping samples, respectively (Fig. 3f). In addition, 5 samples with same fabrication conditions were measured, which showed an average PL lifetime of 541 ± 14 ns for the (PA)_2_PbBr_4_ capping sample and 416 ± 21 ns for the (HDA)PbBr_4_ capping sample, respectively. The largely prolonged PL lifetime also suggested that the trap states of MAPbBr_3_ were largely reduced with the assistance of (PA)_2_PbBr_4_ or (HDA)PbBr_4_ capping layers [46]. The PL lifetime of heterostructures also showed dependence on thickness of 2D perovskite layers (Fig. S10). Both of (PA)_2_PbBr_4_ and (HDA)PbBr_4_ capping samples exhibited the trend of first increasing and then decreasing PL lifetime with the increase in 2D perovskite layer thickness. The trap densities of Ctrl, (PA)_2_PbBr_4_ capping, and (HDA)PbBr_4_ capping samples were evaluated to be 9.03 × 10^10^, 1.48 × 10^10^, and 3.67 × 10^10^ cm^−3^, respectively (Figs. 3g and S11). With capping layers, the trap states of conventional MAPbBr_3_ were largely reduced. The experimental results are in good agreement with the above simulation results, which indicate that the defects are much harder to form in the (PA)_2_PbBr_4_ capping and the (HDA)PbBr_4_ capping samples than in the Ctrl samples.

Time-resolved photocurrent decay (TPC) can reflect the carrier transport properties of materials. TPC of each sample was measured under an electric field of 267 V mm^−1^. The rise time of detectors was 50.07, 12.60, and 18.25 μs for the Ctrl, (PA)_2_PbBr_4_ capping, and (HDA)PbBr_4_ capping samples, respectively (Fig. S12). The fall time of detectors was 202.02, 79.47, and 80.88 μs for the Ctrl, (PA)_2_PbBr_4_ capping, and (HDA)PbBr_4_ capping samples, respectively (Fig. S12). With (PA)_2_PbBr_4_ and (HDA)PbBr_4_ capping layers, the photocurrent decay speed of the detectors increased by approximately threefold, which indicated the carrier transport was significantly accelerated. This is due to the lower defect density at the heterojunction interfaces of (PA)_2_PbBr_4_/MAPbBr_3_ and (HDA)PbBr_4_/MAPbBr_3_. In semiconductors, defects act as traps and recombination centers for charge carriers, hindering their transport. But at these interfaces with reduced defects, carriers move more smoothly and reach the collection electrode faster.

### Performances and Device Structure of Perovskite-Based X-Ray Detectors

**Fig. 4:**
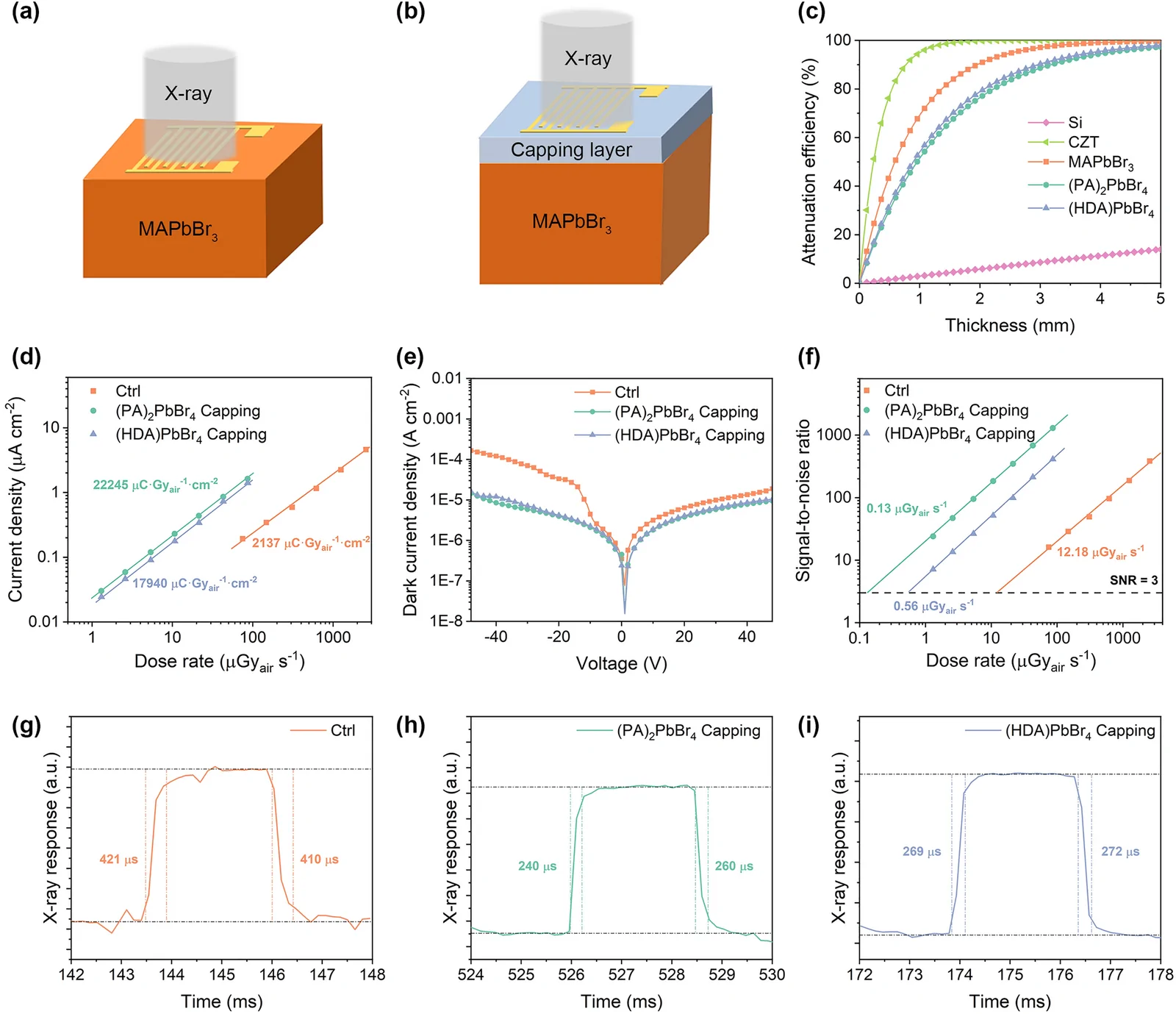
Performances of perovskite-based X-ray detectors. Schematic device structures of **a** detectors based on MAPbBr_3_ without capping layers, and **b** detectors based on MAPbBr_3_ with capping layers. **c** Theoretical attenuation efficiency of MAPbBr_3_, (PA)_2_PbBr_4_, (HDA)PbBr_4_, CdZnTe (CZT), and silicon (Si) for 60 keV X-ray. **d** Sensitivity of Ctrl, (PA)_2_PbBr_4_ capping, and (HDA)PbBr_4_ capping samples. **e** Dark current density versus voltage curves of Ctrl, (PA)_2_PbBr_4_ capping, and (HDA)PbBr_4_ capping samples. **f** Signal-to-noise ratio of Ctrl, (PA)_2_PbBr_4_ capping, and (HDA)PbBr_4_ capping samples. The X-ray response speed of **g** Ctrl, **h** (PA)_2_PbBr_4_ capping, and **i** (HDA)PbBr_4_ capping samples

The X-ray detection performance was evaluated with 2 device configurations of Au/MAPbBr_3_/Au and Au/Capping layer/MAPbBr_3_/Capping layer/Au (Figs. 4a, b and S1). For the Au/MAPbBr_3_/Au configuration, the X-ray was incident along the vertical direction to pass the finger-type Au electrode and MAPbBr_3_ single crystal in sequence (Fig. 4a). For the Au/Capping layer/MAPbBr_3_/Capping layer/Au configuration, the X-ray was incident along the vertical direction to pass the finger-type Au electrode, capping layer, and MAPbBr_3_ single crystal in sequence, of which the capping layer could be (PA)_2_PbBr_4_ or (HDA)PbBr_4_ (Fig. 4b). The (PA)_2_PbBr_4_ capping layer was ~ 300 nm thick, which only absorbed 0.022% of X-ray (Fig. 4c, teal green line). The (HDA)PbBr_4_ capping layer was ~ 450 nm thick, which only absorbed 0.035% of X-ray (Fig. 4c, blue-gray line). For all kinds of samples, the thickness of MAPbBr_3_ single crystal is 1.5 mm, which could absorb 82.938% of the X-ray (Fig. 4c, orange line). Most of the X-ray was absorbed by the MAPbBr_3_ part and the X-ray absorption of capping layers was negligible.

The capping layer greatly improved the performance of MAPbBr_3_-based X-ray detectors. Under 267 V mm^−1^ electric field, all the devices showed a decent linear response to the X-ray within a wide range of dose rates (Fig. 4d). The sensitivity of the (PA)_2_PbBr_4_ capping sample and the (HDA)PbBr_4_ capping sample was 22,245 and 17,940 μC Gy_air_^−1^ cm^−2^, respectively (Fig. 4d, teal green and blue-gray lines). In addition, 5 devices with same fabrication conditions were measured, which showed an average sensitivity of 22,093 ± 2,929 μC Gy_air_^−1^ cm^−2^ for the (PA)_2_PbBr_4_ capping sample and 17,450 ± 2,407 μC Gy_air_^−1^ cm^−2^ for the (HDA)PbBr_4_ capping sample, respectively. However, the sensitivity of the Ctrl sample was only 2,137 μC Gy_air_^−1^ cm^−2^ (Fig. 4d, orange line), which was about 10 times lower than the X-ray detectors with capping layers. The corresponding current–time (*I–t*) curves are shown in Fig. S13, which provide an intuitive view of signal. Besides, the sensitivity of X-ray detectors increased as the electric field increased in the range from 0 V mm^−1^ to around 270 V mm^−1^ (Fig. S14). Then, the sensitivity tended to reach a plateau. Notably, the sensitivity we found experimentally of the (PA)_2_PbBr_4_ capping sample (*S*_e_ = 22,245 μC Gy_air_^−1^ cm^−2^) astonishingly exceeds the theoretical limit *S*_t_ = 16.5 μC Gy_air_^−1^ cm^−2^ by three orders of magnitude (Fig. S15) [47]. This significant discrepancy strongly indicates the presence of photoconductive gain, where the prolonged carrier lifetime amplifies the detector’s sensitivity. The X-ray sensitivity (*S*) is defined as the collected charge (*C*) per unit area (*A*) per unit of radiation exposure (*D*), which presents the efficiency of a detector for converting X-ray to photocurrent (Eq. 8) [48].

8 $$S=\frac{C}{A\times D}= \frac{{Photocurrent \,\,density}}{{Dose \,\,rate}}$$

As our X-ray detector had a photoconductor-type configuration, its sensitivity was highly dependent on its carrier lifetime [6, 49]. Under the high applied electric field, the X-ray excited carriers could move across the circuits for a number of cycles and thus bring in gains to enlarge the sensitivity. This photoconductive gain has a positive correlation to the carrier lifetime [47]. In the previous discussion, the PL lifetime could be regarded as the carrier lifetime. The PL lifetime of the (PA)_2_PbBr_4_ capping sample was 521 ns, which yielded the highest sensitivity among all samples. The PL lifetime of the (HDA)PbBr_4_ capping sample was 411 ns, of which the sensitivity was slightly lower than that of the (PA)_2_PbBr_4_ capping sample. The PL lifetime of the Ctrl sample was only 65 ns, which resulted in the lowest sensitivity.

The dark current was also reduced by adding the capping layers. In the dark environment, the current density–voltage (*J*–*V*) curves of the (PA)_2_PbBr_4_ capping and the (HDA)PbBr_4_ capping samples were below the* J*–*V* curves of the Ctrl sample (Fig. 4e). When the applied voltage was 40 V (electric field = 267 V mm^−1^), the dark current density of the (PA)_2_PbBr_4_ capping sample and the (HDA)PbBr_4_ capping sample was 7.82 × 10^–6^ and 8.59 × 10^–6^ A cm^−2^, respectively. At the same bias, the dark current density of the Ctrl sample was 1.35 × 10^–5^ A cm^−2^, which was about 70% larger than the dark current density of the samples with capping layers. Under a high electric field, the Shockley–Read–Hall (SRH) recombination contributes a major part of current density [18]. With capping layers, the interfacial defects were largely reduced, which decreased the number of SRH recombination centers and caused the decreased dark current density. In addition, the resistivity of (PA)_2_PbBr_4_ and the (HDA)PbBr_4_ was larger than the resistivity of MAPbBr_3_, which could also cause the decreased dark current density [35].

According to the definition of the International Union of Pure and Applied Chemistry, the detection limit is the equivalent dose rate to generate a signal larger than three times of the signal noise [48]. Accordingly, the lowest limit of detection (LoD) was defined as the dose rate yielding a SNR value of 3. With the increased sensitivity and decreased dark current density, the LoD of the (PA)_2_PbBr_4_ capping sample significantly dropped to 0.13 μGy_air_ s^−1^, compared with the LoD (12.18 μGy_air_ s^−1^) of the Ctrl sample (Fig. 4f). On the other hand, the LoD of the (HDA)PbBr_4_ capping sample was 0.56 μGy_air_ s^−1^, which was also much lower than the LoD of the Ctrl sample. The lower LoD implies the detector could work under a lower dose rate, thus reduces the dose for X-ray imaging and damage to human body.

The response speed of detectors is also a key figure of merit for medical imaging [2]. With high response speed, an X-ray image could be captured in a very short time, and thus reduce the total dose of X-ray. The rise time (from 10 to 90%) of Ctrl, (PA)_2_PbBr_4,_ and the (HDA)PbBr_4_ capping samples was 421, 240, and 269 μs, respectively (Fig. 4g–i). The fall time (from 90% to 10%) of Ctrl, (PA)_2_PbBr_4,_ and the (HDA)PbBr_4_ capping samples was 410, 260, and 272 μs, respectively (Fig. 4g–i). With capping layers, the response speed of perovskite-based X-ray detectors could increase to about twice the pristine speed. Besides, these X-ray detectors with capping layers were among the fastest for all reported detectors with different perovskite absorbers (Table S1) [2, 3, 11–13, 20, 48, 50–53]. The fast response speed should attribute to the efficient carrier transport of the heterostructures, which was verified by the TPC measurements in the above part.

### Stability and Ion Movement

**Fig. 5:**
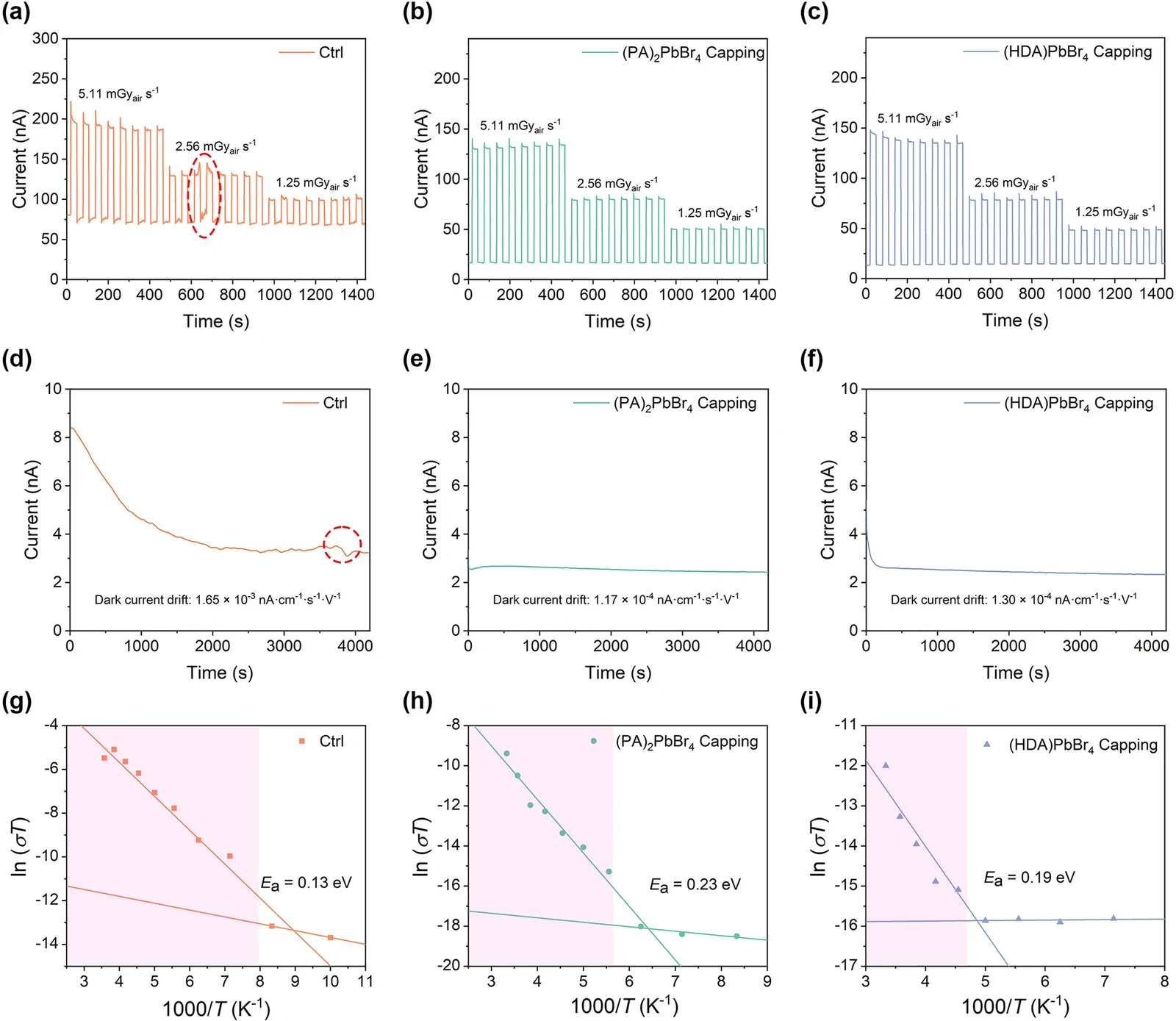
Stability and ion movement. Pulsed X-ray response current curves of **a** Ctrl, **b** (PA)_2_PbBr_4_ capping, and **c** (HDA)PbBr_4_ capping samples. Dark current drift curves of **d** Ctrl, **e** (PA)_2_PbBr_4_ capping, and **f** (HDA)PbBr_4_ capping samples. Temperature-dependent conductivity measurements of **g** Ctrl, **h** (PA)_2_PbBr_4_ capping, and **i** (HDA)PbBr_4_ capping samples

The halide perovskite-based X-ray detectors showed good stability under the electric field of 66.7 V mm^−1^ toward X-ray irradiation (relative humidity: 40 RH%, temperature: 22 °C). All of the detectors were operating under pulsed X-ray exposure with a total dose of 2.15 Gy, which was equivalent to the total dose of over 10^5^ times chest X-ray image (Fig. 5a–c) [4, 54]. After operation, the detector made of MAPbBr_3_ with the (PA)_2_PbBr_4_ capping layer maintained over 99% of the original sensitivity, which was the best among all kinds of detectors (Fig. 5b). Under the same working condition, the detectors made of MAPbBr_3_ with and without the (HDA)PbBr_4_ capping layers maintained > 98% and > 95% of their original sensitivity, respectively (Fig. 5a, c). Besides, the current–time (*I*–*t*) curve of the Ctrl detector showed significantly larger fluctuation than those of the (PA)_2_PbBr_4_ capping and (HDA)PbBr_4_ capping detectors (Fig. 5a–c). From 600 to 730 s, an abnormal increase in current appeared for the Ctrl detector (Fig. 5a, red circle). In addition, both the current with and without X-ray exposure showed an abnormal increase, which indicates the detectors (but not the X-ray source) caused this phenomenon. In stark contrast, the* I*–*t* curves of the (PA)_2_PbBr_4_ capping and (HDA)PbBr_4_ capping detectors showed a square wave-like shape with negligible fluctuation (Fig. 5b, c). In Fig. 5a–c, the stability curves of the three sample groups exhibit overshoot phenomena at the current rising edge, which can be attributed to space charge effects [55]. As commonly observed in defective wide-bandgap semiconductors, the rapid increase in photocurrent at the initial stage of irradiation is due to the collection of a large number of photogenerated carriers. However, some carriers become trapped beneath the electrodes, leading to the formation of space charge. Consequently, an X-ray-induced internal polarization electric field is established, opposing the bias electric field, which reduces charge collection efficiency and causes the photocurrent to decline after reaching its peak. This internal polarization also explains why the photocurrent signal takes several seconds to reach a steady-state value. In contrast, the absence of this phenomenon in Fig. 4g–i is attributed to the excessively short test duration (microsecond scale) for the response time, which is insufficient to establish an effective internal polarization electric field.

To further investigate this phenomenon, the long-term dark currents of different detectors were performed under 6.7 V mm^−1^ electric field. Conspicuous fluctuation could be also observed in the *I*–*t* curve of the Ctrl sample (Fig. 5d, red circle). Considering the ion movement inside MAPbBr_3_, the abnormal fluctuation might be caused by the partially collapse of crystal lattices [56, 57]. Differently, the long-term dark current curves of the (PA)_2_PbBr_4_ capping and (HDA)PbBr_4_ capping detectors were rather smooth (Fig. 5e, f). Besides, a large drop was observed for the Ctrl detector, which resulted in a dark current drift value of 1.65 × 10^–3^ nA cm^−1^ s^−1^ V^−1^ (Fig. 5d). With the (PA)_2_PbBr_4_ and (HDA)PbBr_4_ capping layers, dark current drifts of detectors were remarkably decreased to 1.17 × 10^–4^ and 1.30 × 10^–4^ nA cm^−1^ s^−1^ V^−1^, respectively (Fig. 5e, f). The dark current drift of detectors was higher when under higher electric field. Under 267 V mm^−1^ electric field, dark current drifts were 4.31 × 10^–2^, 9.50 × 10^–4^, and 8.43 × 10^–3^ nA cm^−1^ s^−1^ V^−1^ for the Ctrl, the (PA)_2_PbBr_4_ capping, and (HDA)PbBr_4_ capping detectors, respectively (Fig. S16). Those values were among the lowest reported dark current drifts of halide perovskites, such as CsPbBr_3_ single crystal (1.56 × 10^–3^ nA cm^−1^ s^−1^ V^−1^ under 125 V mm^−1^ electric field), CsPbBr_3_/MAPbBr_3_ heterojunction (3.92 × 10^–4^ nA cm^−1^ s^−1^ V^−1^ under 125 V mm^−1^ electric field), and Cs_0.1_FA_0.85_GA_0.05_Pb(I_0.9_Br_0.1_)_3_:Sr single crystal (8.77 × 10^–5^ nA cm^−1^ s^−1^ V^−1^ under 20 V mm^−1^ electric field) [58, 59].

As halide perovskites are ionic crystals, their ions could migrate driven by the external electrical field [60, 61]. For MAPbBr_3_, the bromide anions (Br^−^) are the major mobile ions, which could result in an ionic current [56]. When the applied electric field continues for a certain time, the ions accumulate at the perovskite–electrode interface, which creates an opposite electric field and decreases the total electric field inside perovskites. In this way, the total current tends to decrease as the operation time increases. All of the *I*–*t* curves measured under dark conditions agreed well with this theory, and the dark current drift values extracted from these curves should relate to the ion migration rate of halide perovskites (Fig. 5d–f) [57]. Thus, the Ctrl sample should have the largest ion migration rate among all samples. The (PA)_2_PbBr_4_ capping sample should have the smallest ion migration rate. The (HDA)PbBr_4_ capping sample should have the medium ion migration rate.

To further confirm the above results, the ion migration activation energy of all samples was measured [13]. In all figures, the temperature-dependent conductivity curves could be divided into two linear regions (Fig. 5g–i). In the high-temperature region, ionic conductivity dominates the total conductivity (Fig. 5g–i, pink region). As the temperature decreases, the ion migration is retarded and causes a fast drop in conductivity. After the temperature decreases to a threshold value, the ion migration is inhibited and electronic conduction becomes the major part (Fig. 5g–i, white region). Applying the Nernst–Einstein relation, the *E*_a_ of ionic conduction could be derived from the high-temperature region, which was used to quantitatively evaluate the ion migration rate. The *E*_a_ of the Ctrl, the (PA)_2_PbBr_4_ capping, and the (HDA)PbBr_4_ capping samples was 0.13, 0.23, and 0.19 eV, respectively. Among them, the (PA)_2_PbBr_4_ capping sample showed the largest activation energy, indicating its ion movement is the hardest and resulting in the slowest ion migration speed. The Ctrl sample showed the smallest activation energy, which resulted in the fastest ion migration speed. Excitingly, these results agreed well with the dark current drift results.

## Conclusion

In summary, a gas-based method was developed to synthesize (PA)_2_PbBr_4_ or (HDA)PbBr_4_ capping layers directly on MAPbBr_3_ single crystals. With the 2D perovskites capping layers, the mobile Br^−^ ions were constrained to the Pb^2+^ ions, which significantly suppresses the ion movement across the lattices and the formation of defects at the interface. In addition, a comparison between the (PA)_2_PbBr_4_ and (HDA)PbBr_4_ capping layers emphasized the importance of steric hindrance of organic layers. With larger steric hindrance of the organic layer, (PA)_2_PbBr_4_ capping samples showed larger ion migration activation energy, less trap density, and longer carrier lifetime, compared with (HDA)PbBr_4_ capping samples. In this way, MAPbBr_3_-based X-ray detectors with the (PA)_2_PbBr_4_ capping layer achieved a sensitivity of 22,245 μC Gy_air_^−1^ cm^−2^, a response speed of 240 μs, and a dark current drift of 1.17 × 10^–4^ nA cm^−1^ s^−1^ V^−1^, while the MAPbBr_3_-based X-ray detectors without capping layers only showed a sensitivity of 2,137 μC Gy_air_^−1^ cm^−2^, a response speed of 421 μs, and a dark current drift of 1.65 × 10^–3^ nA cm^−1^ s^−1^ V^−1^. Overall, this study presents a precise strategy to synthesize nanoscale 2D perovskite directly on 3D perovskite, provides insights into the structure-dependent properties of perovskite-based heterojunctions, and realizes high-performance X-ray detectors for future applications.

## Supplementary Information

Below is the link to the electronic supplementary material.

Supplementary file1 (DOCX 10855 KB)
