# Supplementary material for: Gas-Phase Construction of Compact Capping Layers for High-Performance Halide Perovskite X-Ray Detectors
Source: Nanomicro Lett. 2025 Dec 20;18:89. doi: 10.1007/s40820-025-01900-3 (PMC12717332; doi:10.1007/s40820-025-01900-3)
Supplement: Supplementary file 1 — Supplementary file1 (DOCX 10855 KB) [file 40820_2025_1900_MOESM1_ESM.docx]

Supporting Information for

**Gas Phase Construction of Compact Capping Layers for High-Performance Halide Perovskite X-Ray Detectors**

Bin Zhang^1, 2,^ †, Chuanyun Hao^1,^ †, Shoufeng Zhang^3,^ †, Bin Xue^1^, Xiangfan Xie^1^, Shengqiao Zeng^1^, Bin Yang^1^, Fang Xu^1^, Hui Li^1^, Xin’an Zhang^1^, Zhang Qu^1^, Kaihang Ye^4^, Guangda Niu^5, 6^, Wallace C. H. Choy^7^, Kezhou Fan^8^, Kam Sing Wong^8^, Lei Yan^2^, Xingzhu Wang^2,^ *, Shuang Xiao^1,^ *, Cangtao Zhou^1^

^1^ Shenzhen Key Laboratory of Ultraintense Laser and Advanced Material Technology, Center for Intense Laser Application Technology (iLaT) and College of Engineering Physics, Shenzhen Technology University, Shenzhen 518118, P. R. China

^2^ Engineering and Research Center for Integrated New Energy Photovoltaics & Energy Storage Systems of Hunan Province and School of Electrical Engineering, University of South China, Hengyang 421001, P. R. China

^3^ School of Electrical Engineering, Guangxi University of Science and Technology, Liuzhou 545006, P. R. China

^4^ Institute for Sustainable Transformation School of Chemical Engineering and Light Industry, Guangdong University of Technology, Guangzhou 510006, P. R. China

^5^ Research Institute of Huazhong University of Science and Technology in Shenzhen, Shenzhen 518052, P. R. China

^6^ Wuhan National Laboratory for Optoelectronics, Huazhong University of Science and Technology, Wuhan 430074, P. R. China

^7^ Department of Electrical and Electronic Engineering, The University of Hong Kong, Pokfulam Road, Hong Kong SAR, P. R. China

^8^ Department of Physics and William Mong Institute of Nano Science and Technology, The Hong Kong University of Science and Technology, Clear Water Bay, Kowloon, Hong Kong S.A.R., P. R. China

†Bin Zhang, Chuanyun Hao, and Shoufeng Zhang contributed equally to this work.

*Corresponding authors. E-mail: [2022000041@usc.edu.cn](mailto:2022000041@usc.edu.cn) (Xingzhu Wang); [xiaoshuang@sztu.edu.cn](mailto:xiaoshuang@sztu.edu.cn) (Shuang Xiao)

**DFT Calculations**

The first-principles calculations were performed using the Vienna Ab initio Simulation Package (VASP, version5.4.4) [S1, S2] with standard projector augmented-wave (PAW) method and the exchange-correlation functional for generalized gradient approximation (GGA) in PBE format [S1]. The energy cut-off was set as 450 eV with energy convergence criterion of 10^-5^ eV and force converged accuracy of 0.01 eV/Å. A 2🞨3🞨3 *k*-points mesh was set in the Brillouin zone for all DFT calculations. The DFT-D3(Grimme 2006) [S2] was used to describe the vdW-interaction of organic-inorganic interfaces. The post-processing of the energy calculations was obtained by VASPKIT toolkit.

**Supplementary Figures and Tables**


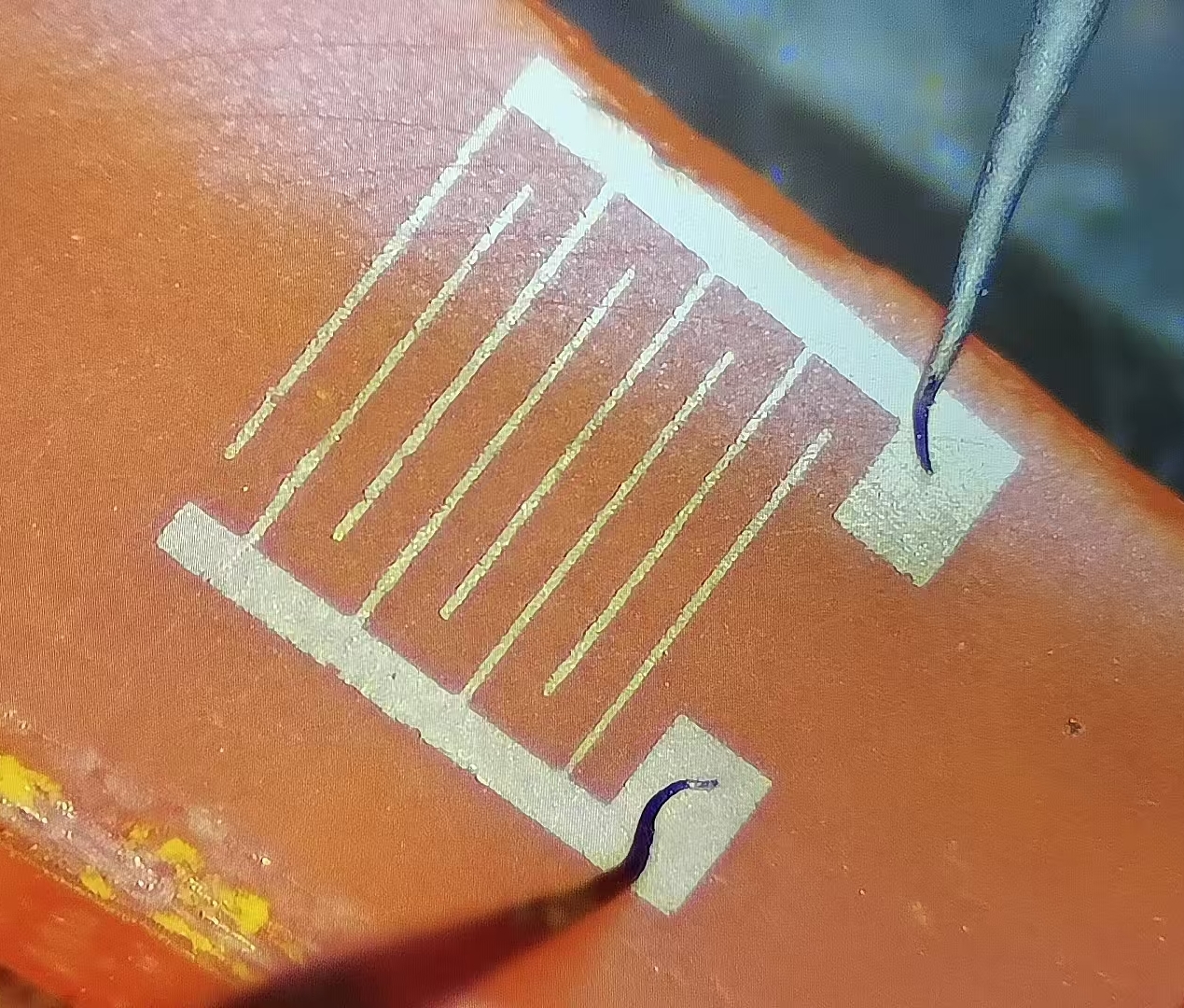


**Fig. S1** The photograph of the X-ray detector with lateral electrodes


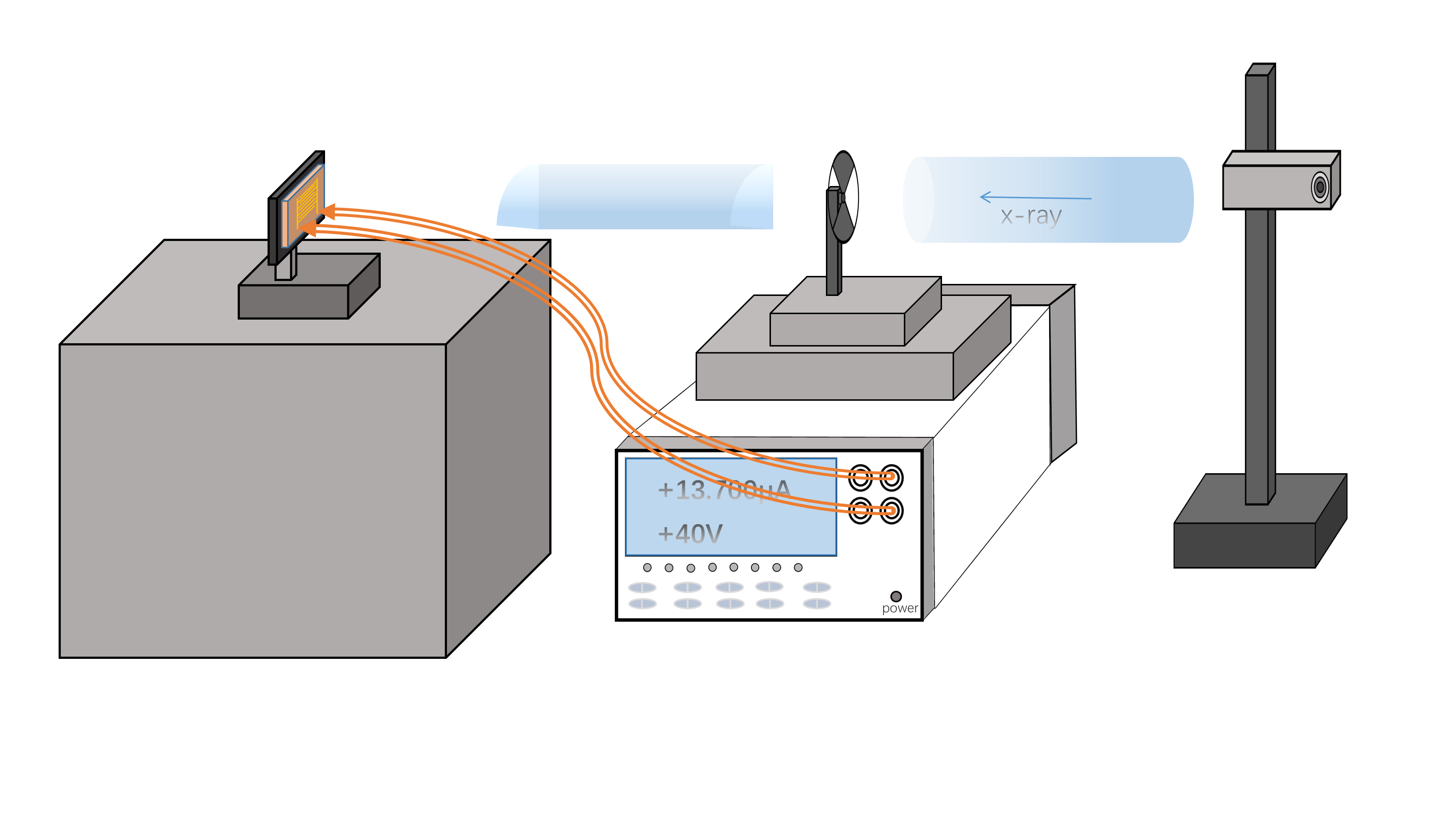


**Fig. S2** Schematic illustration of the X-ray response speed measurement


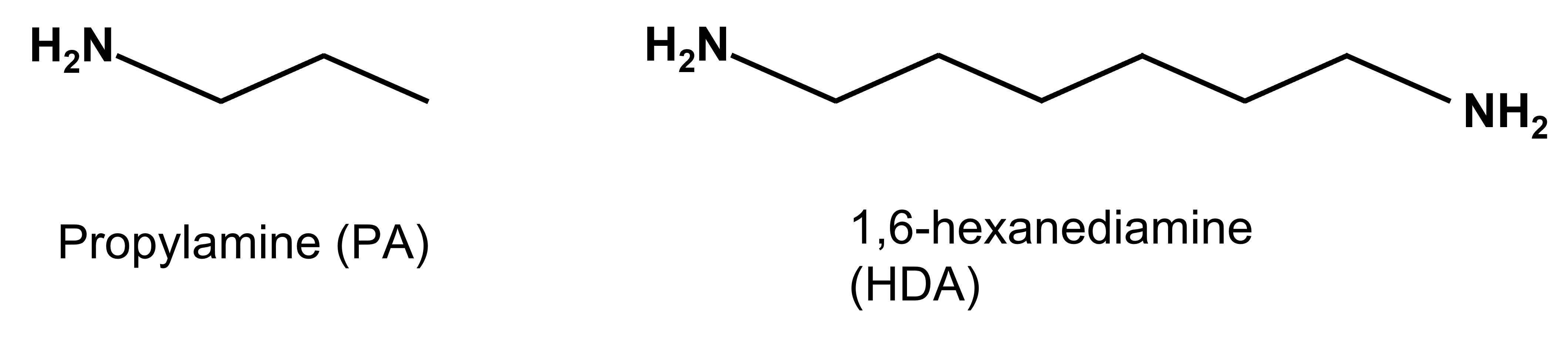


**Scheme S1** The schematic structure of propylamine (PA) and 1,6-hexanediamine (HDA)


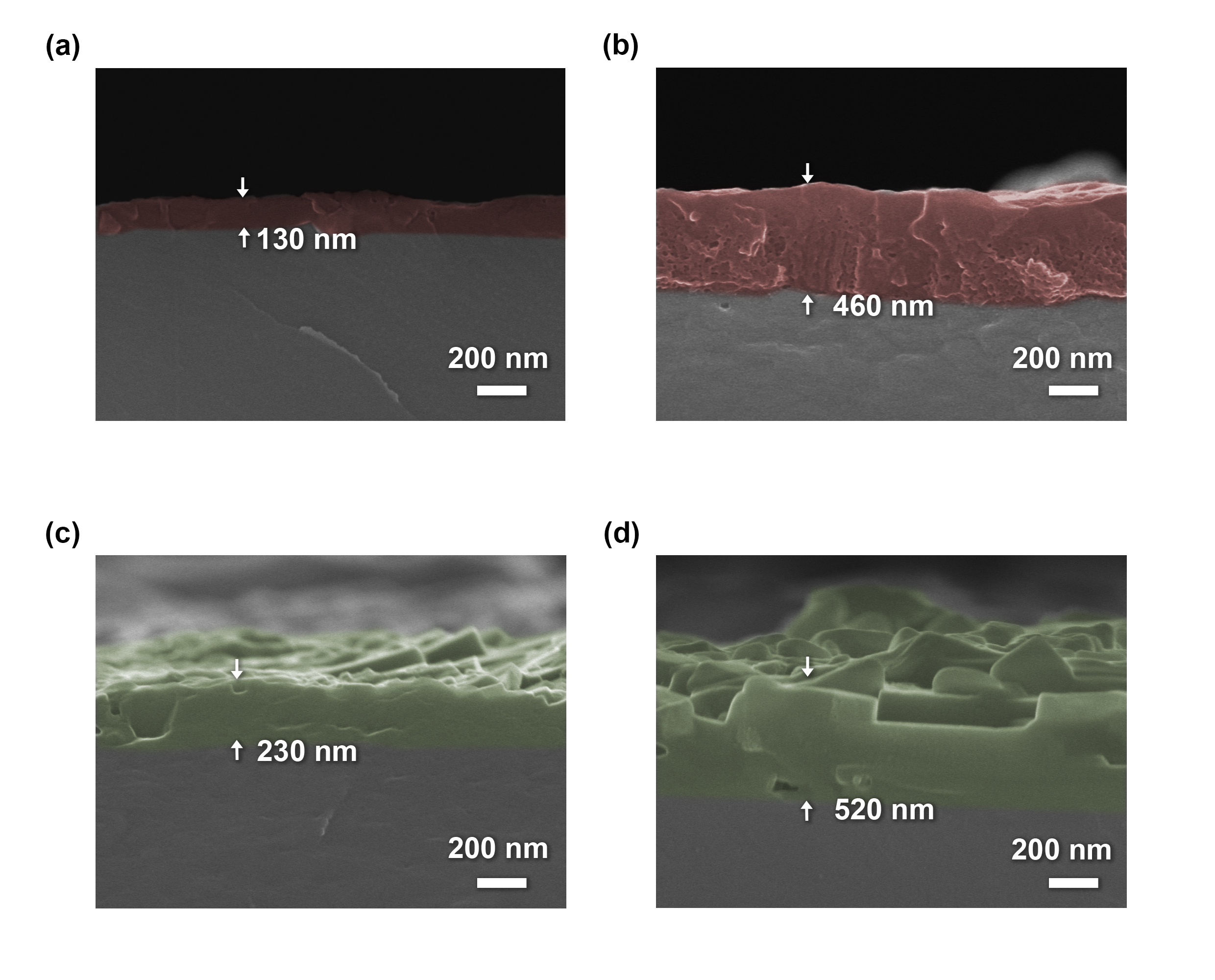


**Fig. S3** Side-view scanning electron microscope (SEM) images of the MAPbBr_3_ single crystal with the (PA)_2_PbBr_4_ capping layers: (**a**) 130 nm (PA)_2_PbBr_4_ capping layer (PA 130) and (**b**) 460 nm (PA)_2_PbBr_4_ capping layer (PA 460). (PA)_2_PbBr_4_ capping layers are in red. SEM images of the MAPbBr_3_ single crystal with the (HDA)PbBr_4_ capping layers: (**c**) 230 nm (HDA)PbBr_4_ capping layer (HDA 230) and (**d**) 520 nm (HDA)PbBr_4_ capping layer (HDA 520). (HDA)PbBr_4_ capping layers are in green


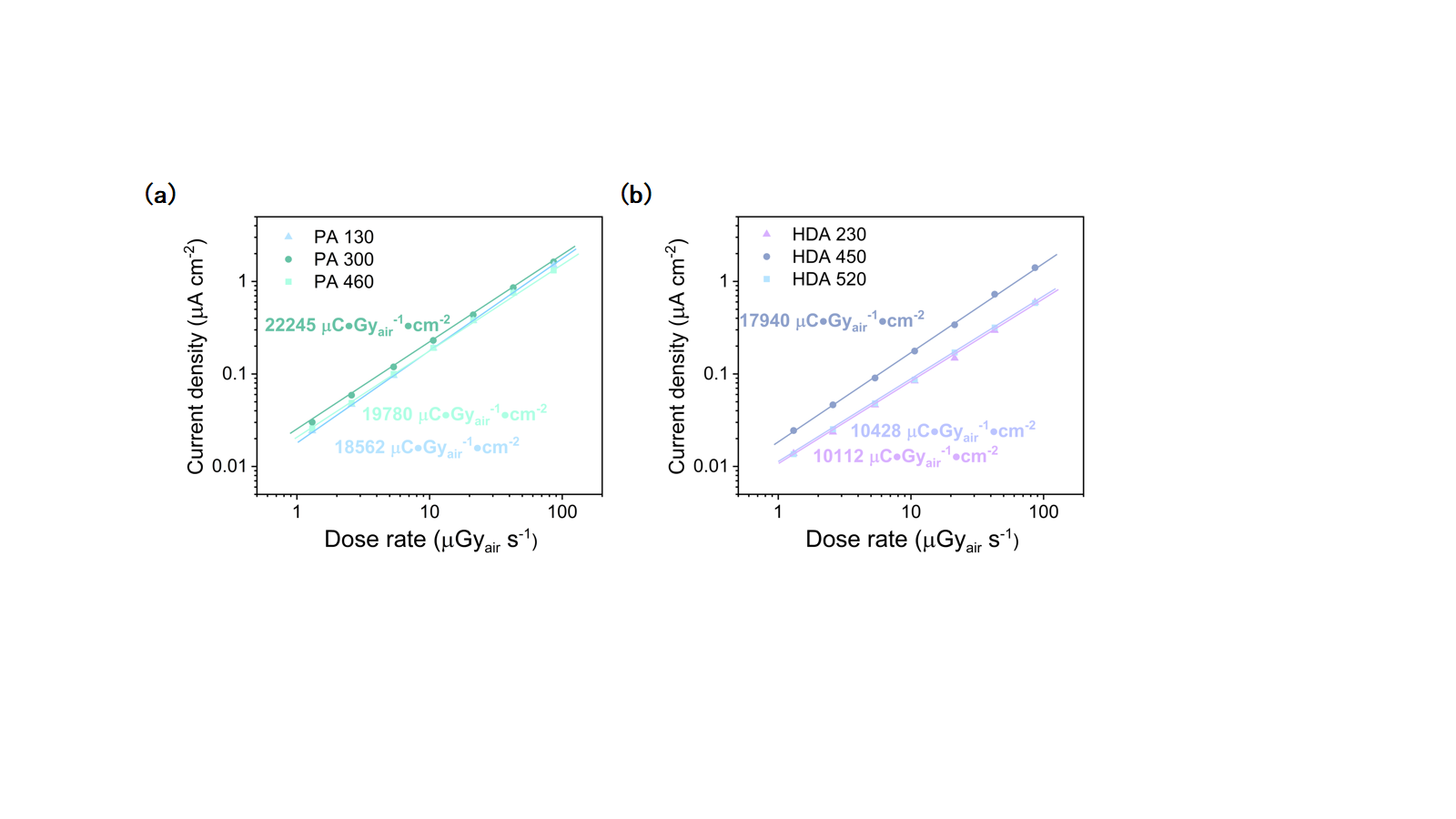
**Fig. S4** Sensitivity of devices with varying 2D perovskite layer thicknesses. (**a**) (PA)_2_PbBr_4_ capping devices: PA 130, PA 300, PA 460 denote 2D perovskite (PA)_2_PbBr_4_ thicknesses of 130 nm, 300 nm, and 460 nm, respectively. (**b**) (HDA)PbBr_4_ capping devices: HDA 230, HDA 450, HDA 520 correspond to 2D perovskite thicknesses of 230 nm, 450 nm, and 520 nm, respectively


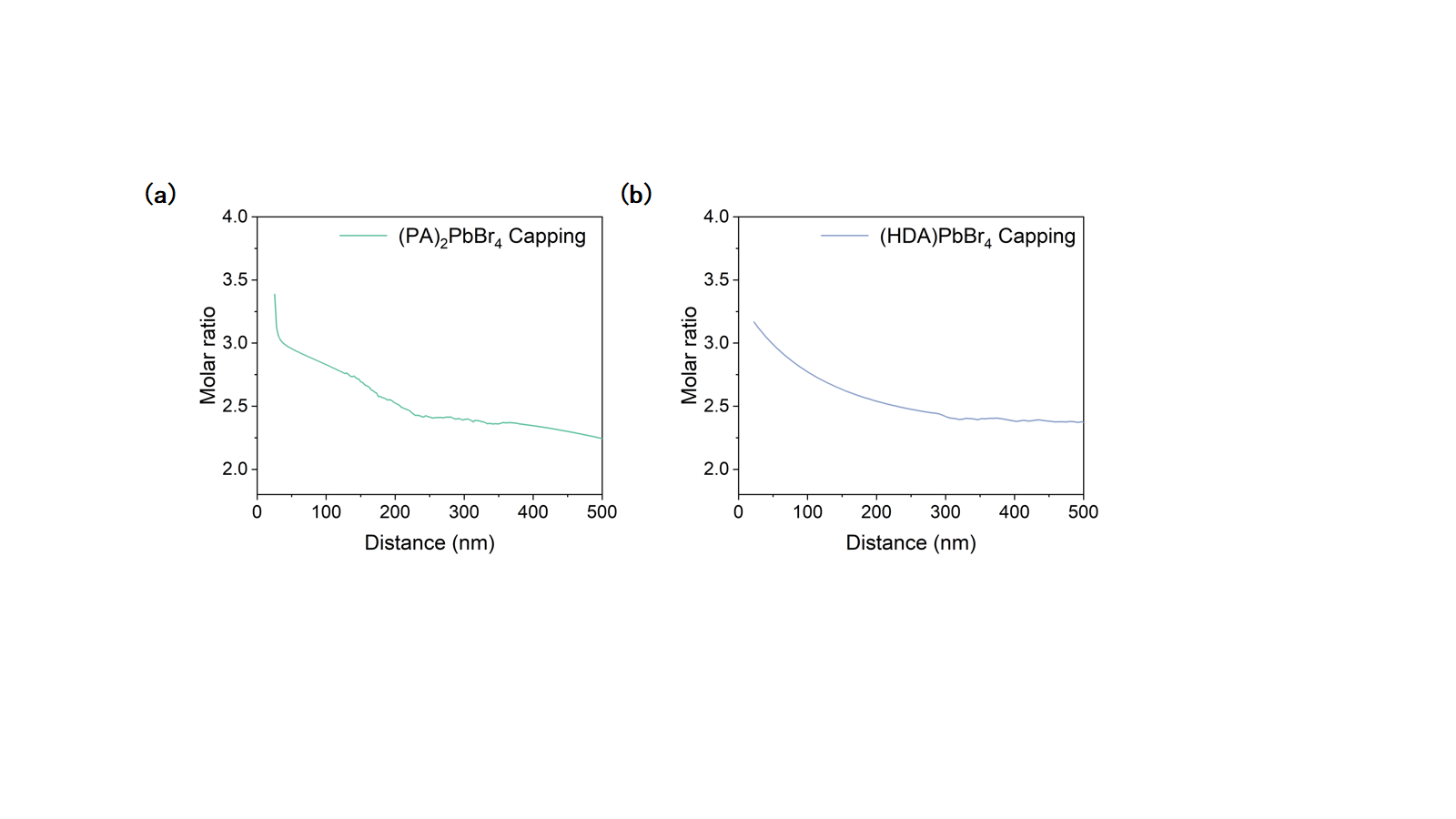


**Fig. S5** Elements molar ratios of Br/Pb alone the cross-section of (**a**) the (PA)_2_PbBr_4_ capping and (**b**) (HDA)PbBr_4_ capping samples


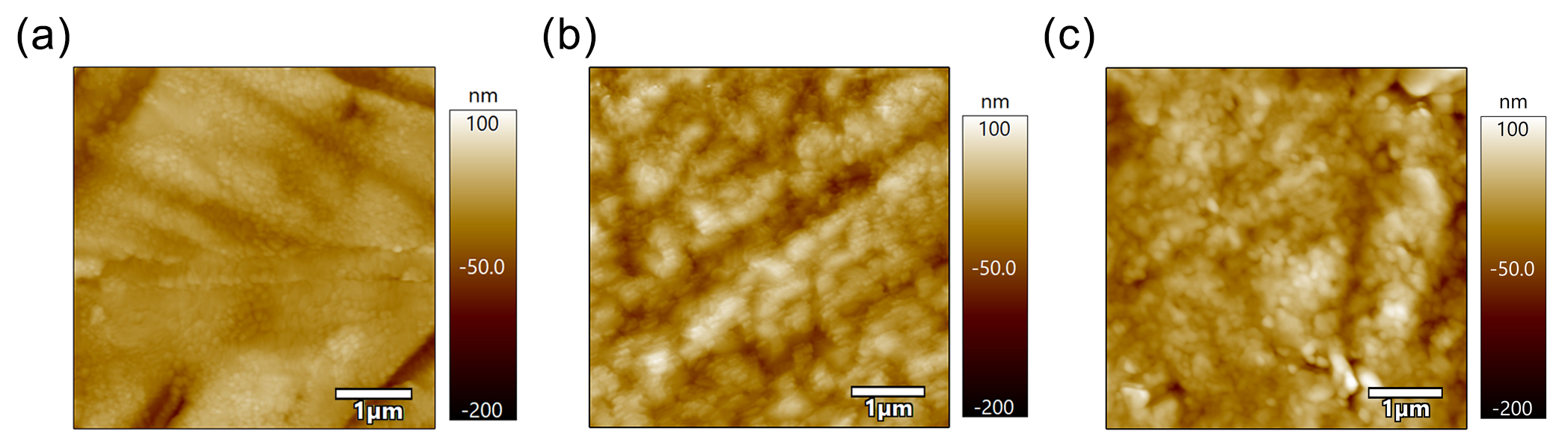


**Fig. S6** Top-view atomic force microscopy (AFM) height images of (**a**) Ctrl, (**b**) (PA)_2_PbBr_4_ capping, and (**c**) (HDA)PbBr_4_ capping samples


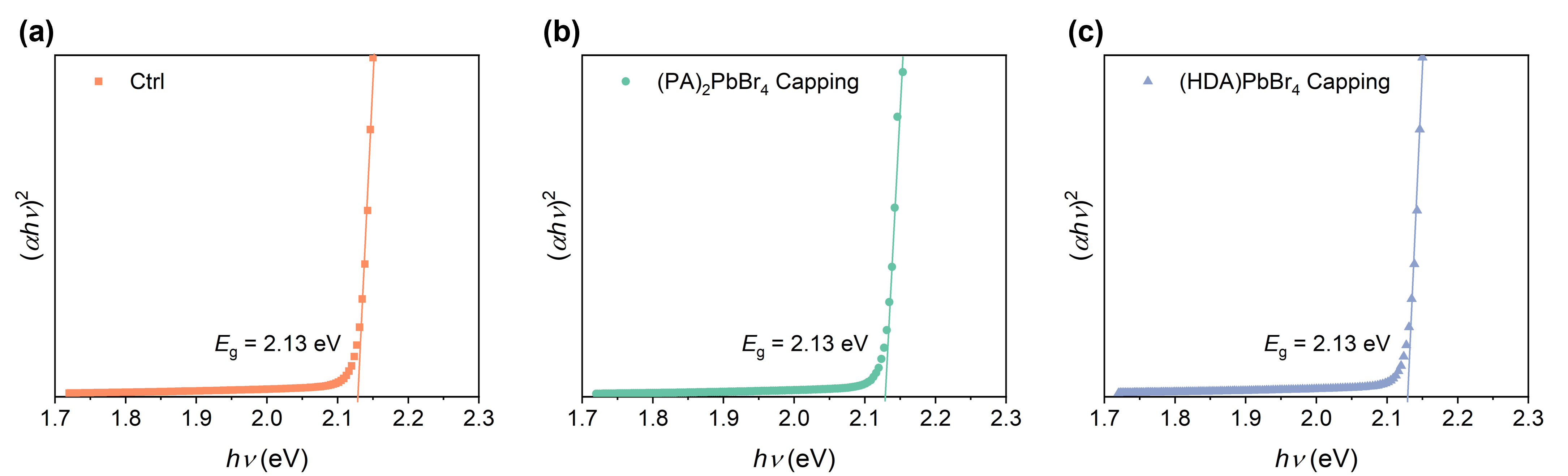


**Fig. S7** Taut plots of (**a**) Ctrl, (**b**) (PA)_2_PbBr_4_ capping, (**c**) (HDA)PbBr_4_ capping samples. The thicknesses of the two-dimensional perovskites (PA)_2_PbBr_4_ and (HDA)PbBr_4_ are both less than 500 nm. Compared with the several-millimeter-thick three-dimensional perovskite MAPbBr_3_, their influence on the UV-Vis spectroscopy test is basically negligible. Therefore, the displayed 2.13 eV bandgap corresponds to that of the three-dimensional perovskite MAPbBr_3_


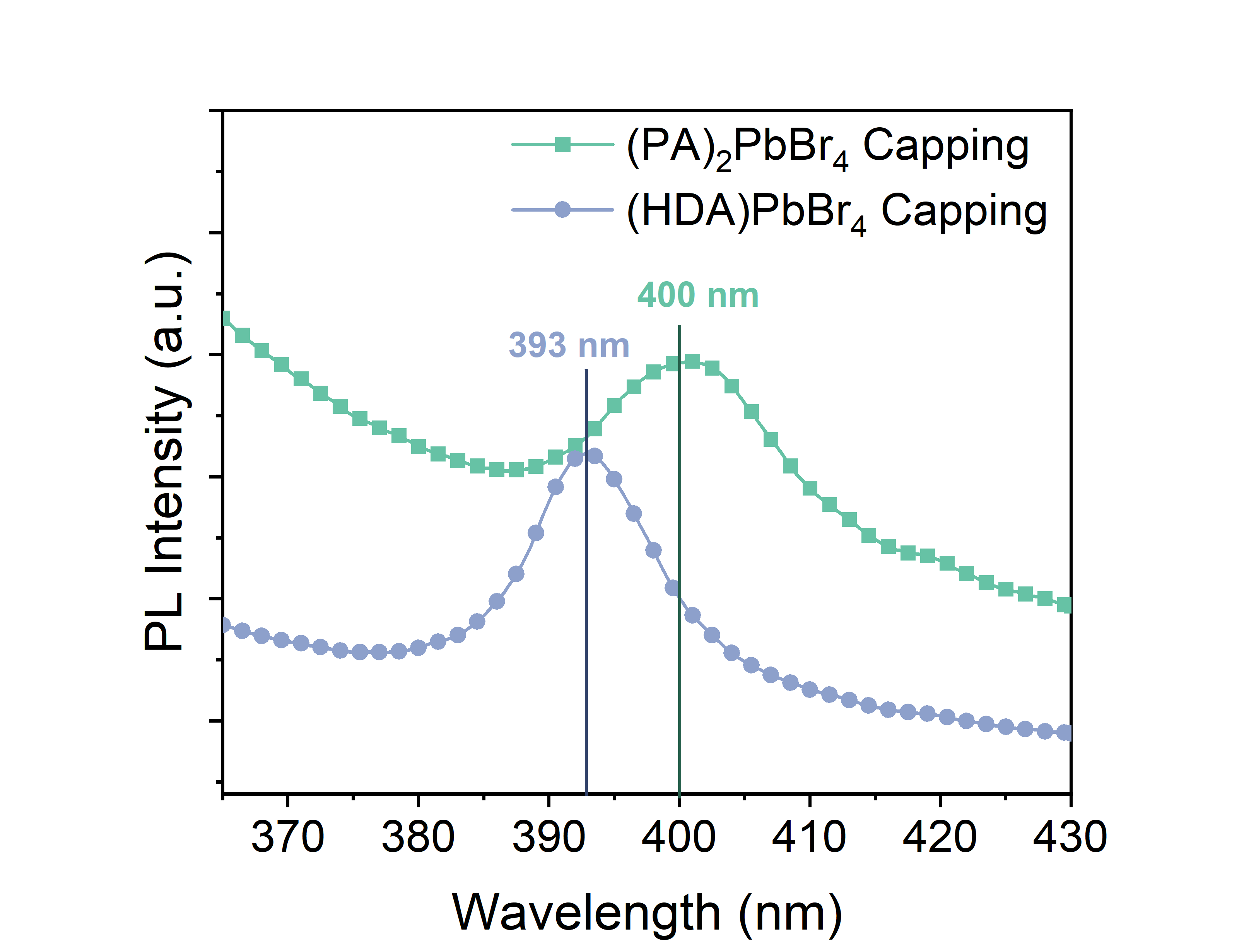


**Fig. S8** Steady-state photoluminescence of (PA)_2_PbBr_4_ capping and (HDA)PbBr_4_ capping samples


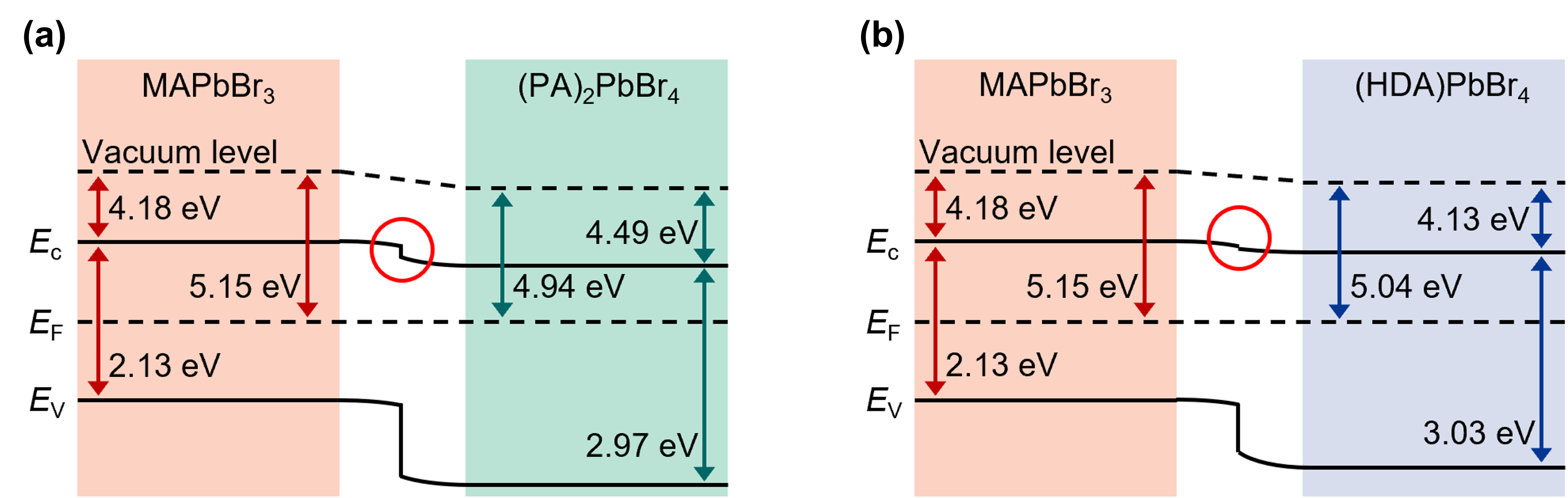


**Fig. S9** Schematic band alignment of (**a**) the MAPbBr_3_-(PA)_2_PbBr_4_ heterojunction and (**b**) the MAPbBr_3_-(HDA)PbBr_4_ heterojunction, which show the energy levels of materials at equilibrium. The energy drop at interfaces are highlighted by red circles


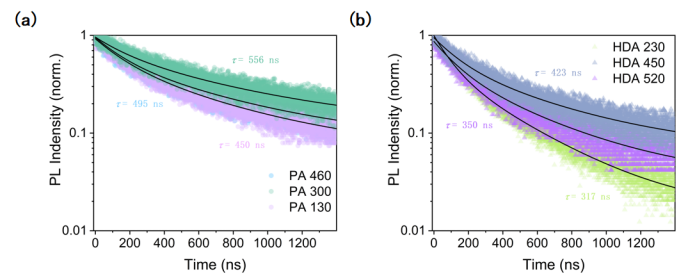


**Fig. S10** TRPL of heterostructures with varying 2D perovskite layer thicknesses. (**a**) (PA)₂PbBr₄ capping samples: PA 130, PA 300, PA 460 denote 2D perovskite (PA)₂PbBr₄ thicknesses of 130 nm, 300 nm, and 460 nm, respectively. (**b**) (HDA)PbBr₄ capping samples: HDA 230, HDA 450, HDA 520 correspond to 2D perovskite thicknesses of 230 nm, 450 nm, and 520 nm


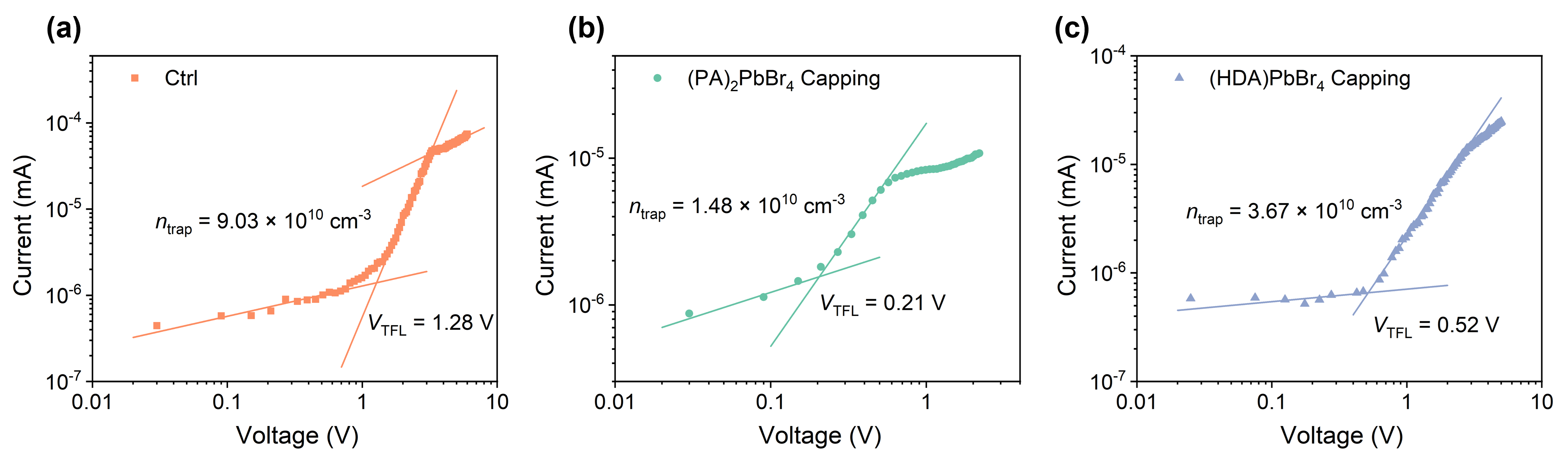


**Fig. S11** Space charge limited current (SCLC) of (**a**) Ctrl, (**b**) (PA)_2_PbBr_4_ capping, and (**c**) (HDA)PbBr_4_ capping samples


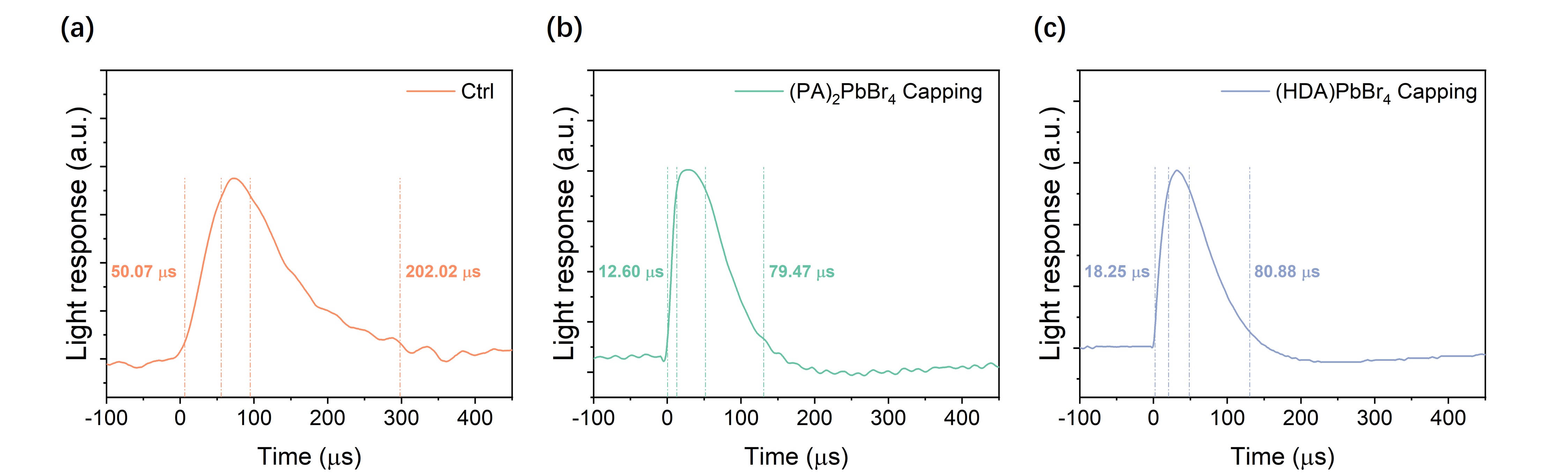


**Fig. S12** The Time-Resolved Photocurrent Decay curves of (**a**) Ctrl, (**b**) (PA)_2_PbBr_4_ capping, and (**c**) (HDA)PbBr_4_ capping samples. The electric field for measurements was 267 V mm^-1^


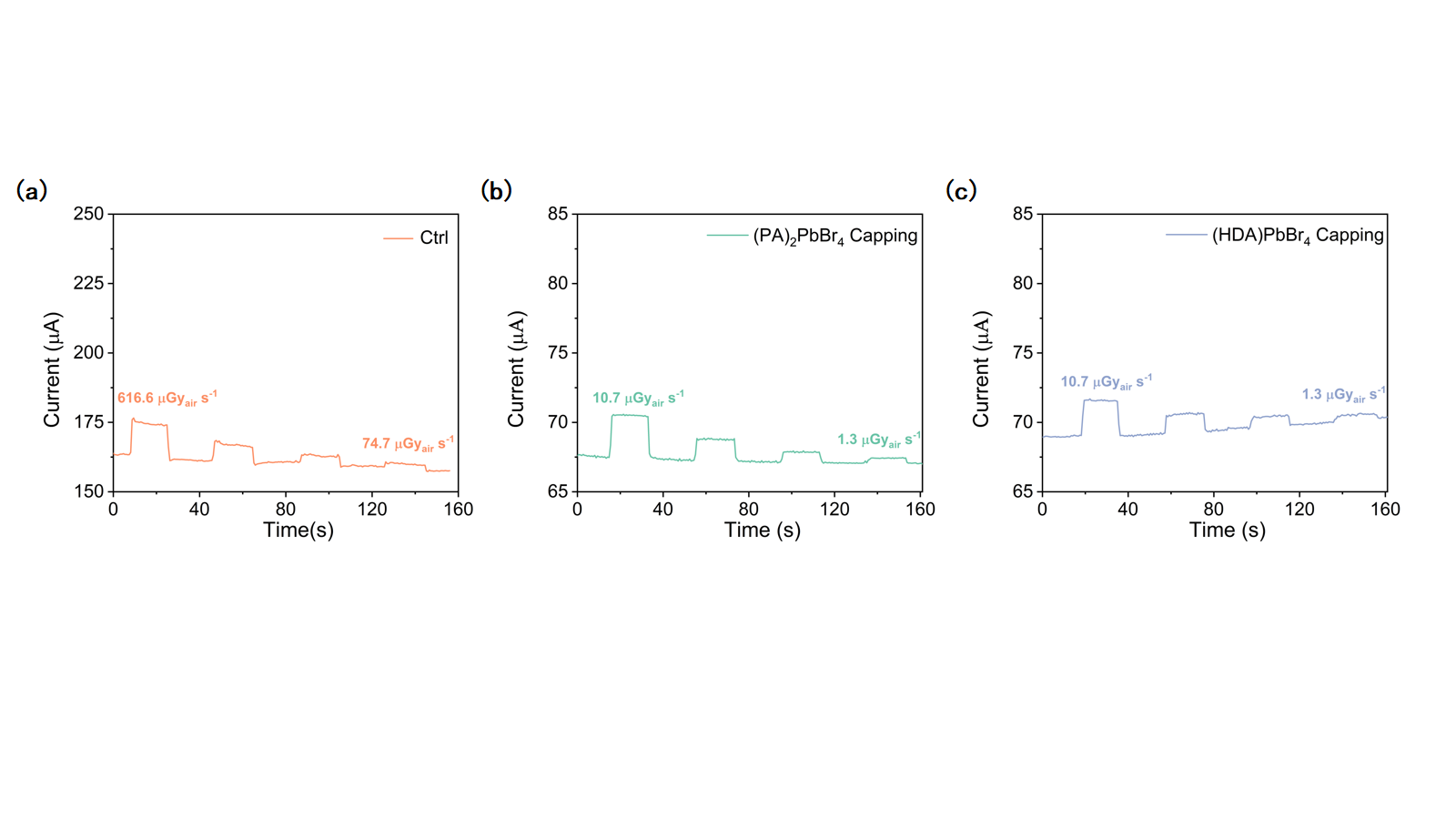


**Fig. S13** (**a**) The current-time (*I*-*t*) curve of the Ctrl device under X-ray dose rates ranging from 74.7 to 616.6 μGy_air_ s^-1^. *I*-*t* curves of (**b**) the (PA)_2_PbBr_4_ capping and (**c**) the (HDA)PbBr_4_ capping devices under X-ray dose rates ranging from 1.3 to 10.7 μGy_air_ s^-1^


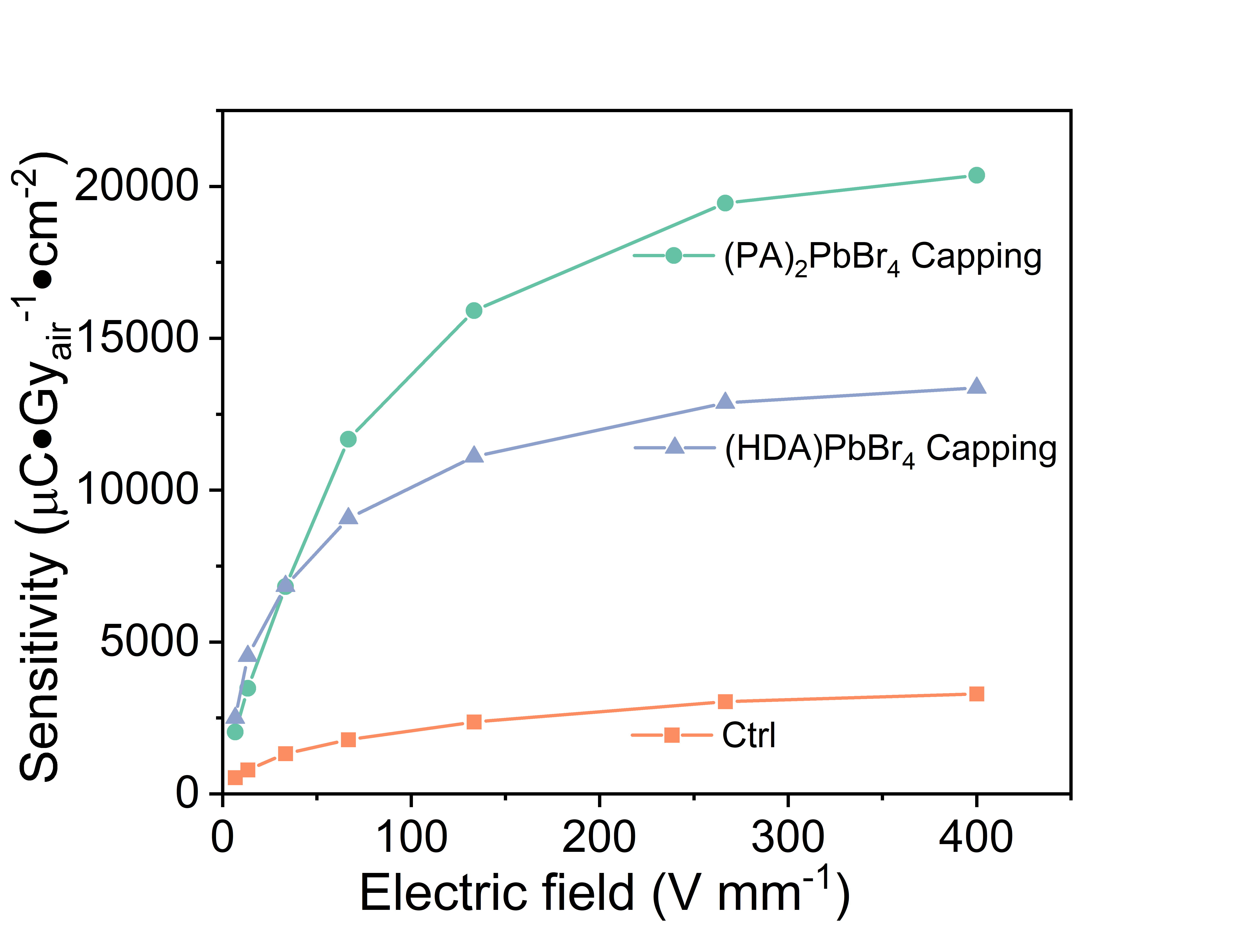


**Fig. S14** Sensitivity of Ctrl, (PA)_2_PbBr_4_ capping, and (HDA)PbBr_4_ capping samples under different electric fields. The dose rate of X-ray for measurements of the Ctrl sample was 5111.11 μGy_air_ s^-1^. The dose rate of X-ray for measurements of the (PA)_2_PbBr_4_ capping and (HDA)PbBr_4_ capping samples was 86.11 μGy_air_ s^-1^


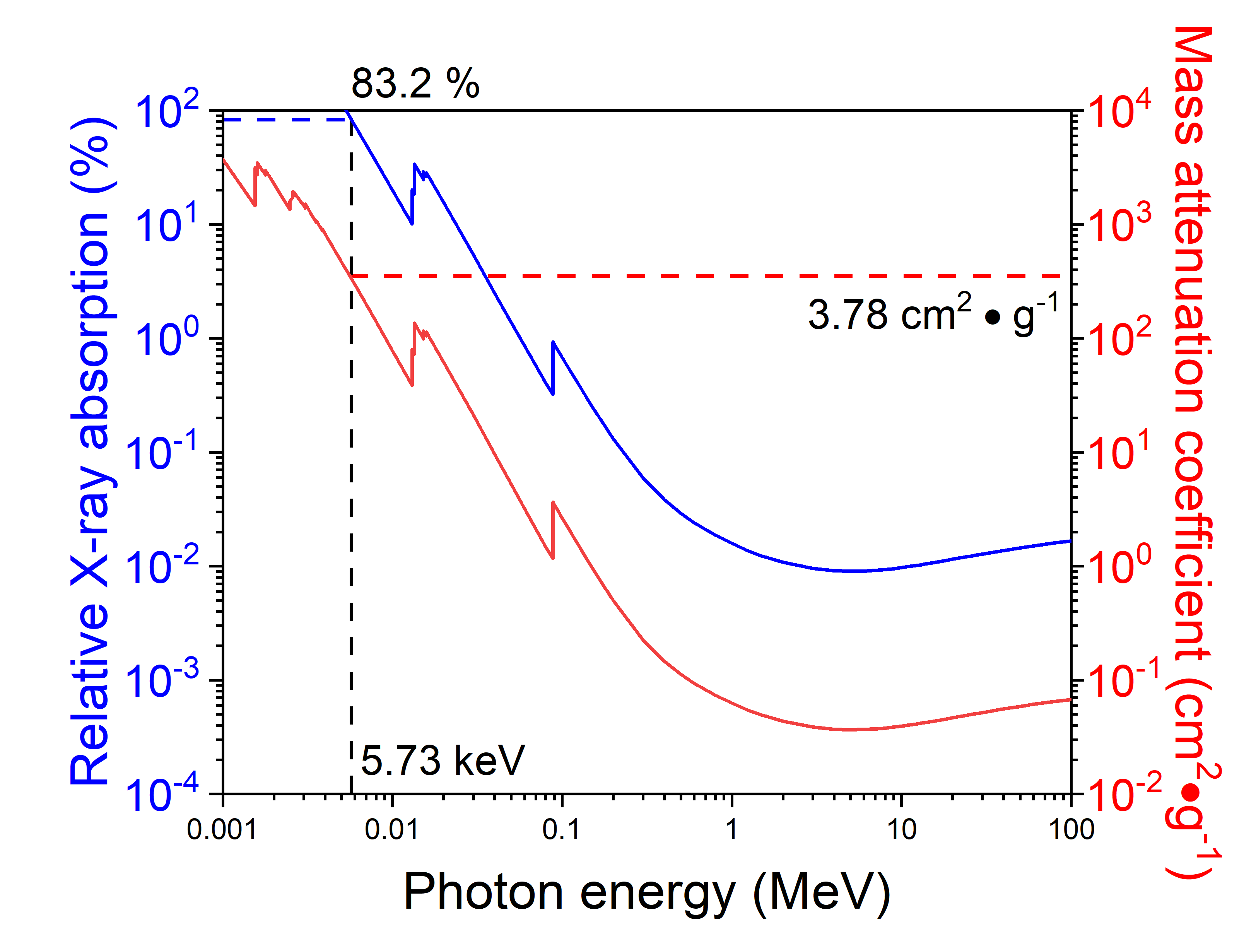


**Fig. S15** Relative X-ray absorption (blue curve) and mass attenuation coefficient (red curve) of MAPbBr_3_ in the 1 eV ~ 100 keV energy range. The relative X-ray absorption coefficient (𝜖) was directly obtained from *μ*_P_ by applying the relationship: 𝜖 = 1 – exp(–*μ*_P_·*ρd)*. By considering a photon energy *E_ph_* = 5.73 keV, which is the most probable energy in the case of a Cu-target X-ray tube, the mass attenuation coefficient is *μ_P_* = 3.78 cm^2^ •g^-1^, corresponding to a relative X-ray absorption 𝜖 = 83.2% for a 1.5 mm-thick crystal. At the same photon energy (5.73 keV), the mass attenuation coefficient of dry air [5] is *μ* = 9.92 cm^2^ g^-1^. Finally, being the bandgap energy *E_g_* = 2.13 eV, the estimated electron-hole pair creation energy is *E_i_* = 2 × 2.13 + 1.43 = 5.69 eV.

By inserting all the numerical values into the *S*_t_ equation we obtain:

$$S_{t}=\frac{e\mu_{P}\rho d}{\mu E_{i}}=16.5 \mu C\cdot{Gy_{air}}^{-1}\cdot\mathrm{cm}^{-2}$$

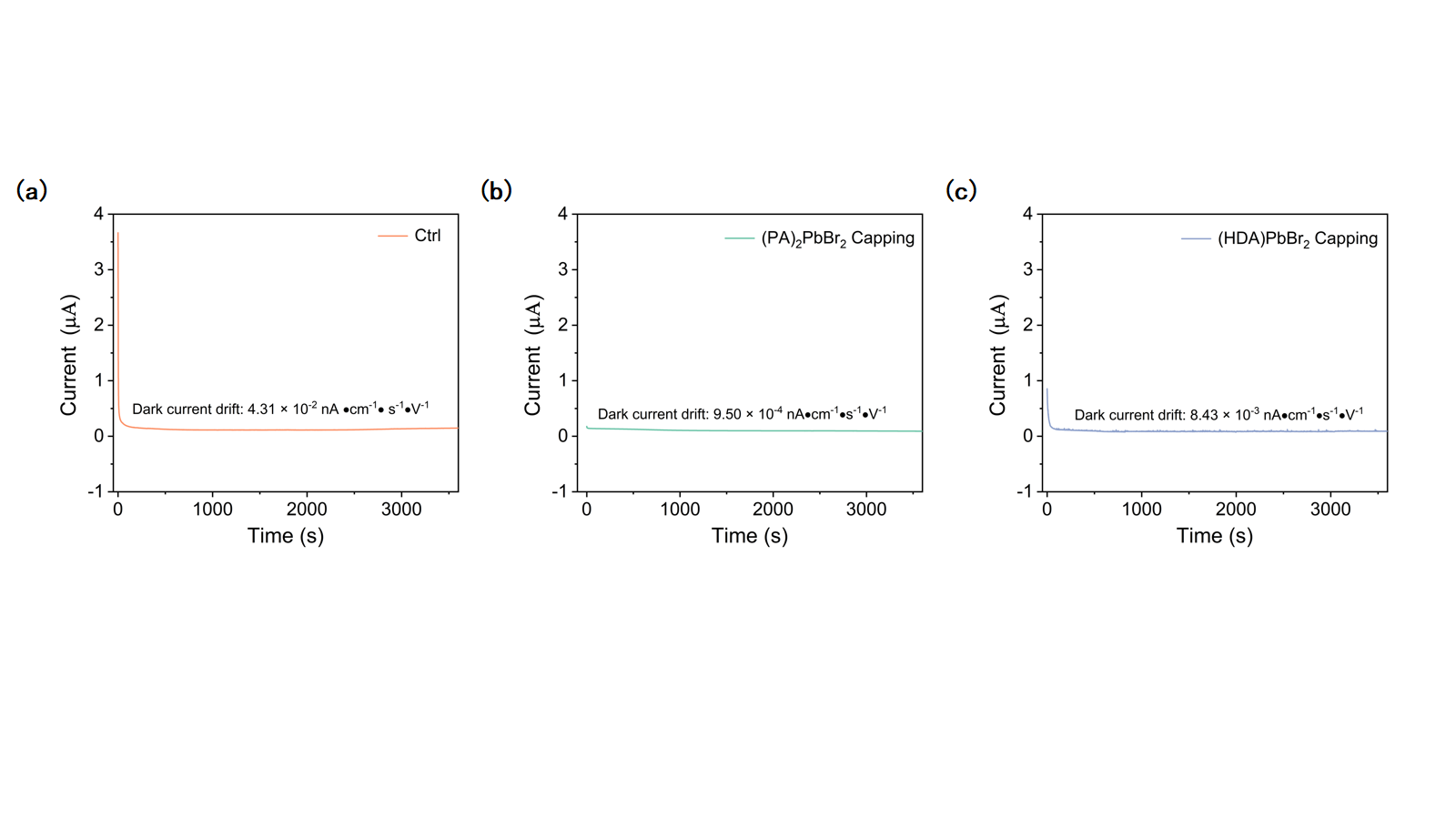


**Fig. S16** The long-term dark current drift of (**a**) Ctrl, (**b**) (PA)_2_PbBr_4_ capping, and (**c**) (HDA)PbBr_4_ capping samples under an electric field of 267 V mm^-1^

**Table S1** Performance comparison of perovskite-based X-ray detectors

| Materials | Electric field  (V mm^-1^) | X-ray  energy  (kVp) | Sensitivity  (μC·Gy_air_^-1^·cm^-2^) | LoD  (μGy_air_ s^-1^) | Response time  (ms) | References |
| --- | --- | --- | --- | --- | --- | --- |
| MAPbBr_3_ | 0.1 | 50 | 80 | 0.5 | 0.73 | [S6] |
| MAPbBr_3_ | 2.67 | 40 | 7275 | 0.67 | / | [S7] |
| MAPbBr_3_ | 7.1 | 40 | 2181 | 0.48 | / | [S8] |
| FAPbI_3_ | 80 | 70 | 4.15 × 10^5^ | 0.001 | 0.214 | [S9] |
| MA_0.42_FA_0.58_PbI_3_ | 71 | 22 | 1.16 × 10^6^ | 0.037 | 20000 | [S10] |
| Cs_0.05_FA_0.9_MA_0.05_PbI_3_ | 200 | 59.3 | 1.5 × 10^4^ | / | 0.235 | [S11] |
| CsPbI_2_Br | 125 | 50 | 1.48 × 10^5^ | 0.28 | / | [S12] |
| CsPbBr_3_ | 21 | 50 | 5.57 × 10^4^ | 0.215 | 92 | [S13] |
| (BDA)PbI_4_ | 310 | 40 | 242 | 0.43 | 7.3 | [S14] |
| 2D layered  (NH_4_)_3_Bi_2_I_9_ | 10 | 50 | 8200 | 0.055 | 80 | [S15] |
| MA_3_Bi_2_I_9_ | 60 | 40 | 1947 | 0.083 | 23 | [S16] |
| MAPbBr_3_ - MAPbI_3_ | 80 | 40 | 3.98 × 10^5^ | 0.012 | 1.5 | [S17] |
| MAPbI_3_ - (PEA)_2_MA_3_Pb_4_I_13_ | 33.3 | 70 | 1.95 × 10^4^ | 0.48 | / | [S18] |
| MAPbBr_3_ -(PA)_2_PbBr­_4_ | 267 | 60 | 2.22 × 10^4^ | 0.13 | 0.240 | This work |
| MAPbBr_3_ -(HDA)PbBr­_4_ | 267 | 60 | 1.79 × 10^4^ | 0.56 | 0.269 | This work |

**Supplementary References**

1. G. Kresse, J. Furthmüller, Efficiency of ab-initio total energy calculations for metals and semiconductors using a plane-wave basis set. Comput. Mater. Sci. **6**(1), 15–50 (1996). <https://doi.org/10.1016/0927-0256(96)00008-0>
2. G. Kresse, J. Furthmüller, Efficient iterative schemes for *ab initio* total-energy calculations using a plane-wave basis set. Phys. Rev. B **54**(16), 11169–11186 (1996). <https://doi.org/10.1103/physrevb.54.11169>
3. J. Perdew, K. Burke, M. Ernzerhof, Generalized gradient approximation made simple. Phys. Rev. Lett. **77**(18), 3865–3868 (1996). <https://doi.org/10.1103/PhysRevLett.77.3865>
4. S. Grimme, J. Antony, S. Ehrlich, H. Krieg, A consistent and accurate *ab initio* parametrization of density functional dispersion correction (DFT-D) for the 94 elements H-Pu. J. Chem. Phys. **132**(15), 154104 (2010). <https://doi.org/10.1063/1.3382344>
5. J. H. Hubbell, S. M. Seltzer, X-Ray Mass Attenuation Coefficients, NIST Standard Reference Database 126 (NISTIR 5632), accessed: October, 2023.
   <https://dx.doi.org/10.18434/T4D01F>
6. H. Wei, Y. Fang, P. Mulligan, W. Chuirazzi, H.-H. Fang et al., Sensitive X-ray detectors made of methylammonium lead tribromide perovskite single crystals. Nat. Photonics **10**(5), 333–339 (2016). <https://doi.org/10.1038/nphoton.2016.41>
7. D. Liu, L. Jiang, X. Jiang, X. Sun, G. Zhang et al., Interface-tension-assisted temperature-gradient crystallization of high-quality MAPbBr_3_ perovskite single crystals with low defect densities. ACS Appl. Mater. Interfaces **15**(49), 57846–57855 (2023). <https://doi.org/10.1021/acsami.3c13614>
8. Z. Zhu, W. Li, W. Deng, W. He, C. Yan et al., Controllable metastable growth of perovskite single crystals for highly sensitive X-ray detection. J. Mater. Chem. C **10**(17), 6837–6845 (2022). <https://doi.org/10.1039/D2TC00235C>
9. D. Chu, B. Jia, N. Liu, Y. Zhang, X. Li et al., Lattice engineering for stabilized black FAPbI_3_ perovskite single crystals for high-resolution X-ray imaging at the lowest dose. Sci. Adv. **9**(35), eadh2255 (2023). <https://doi.org/10.1126/sciadv.adh2255>
10. W.-G. Li, X.-D. Wang, Y.-H. Huang, D.-B. Kuang, Ultrasound-assisted crystallization enables large-area perovskite quasi-monocrystalline film for high-sensitive X-ray detection and imaging. Adv. Mater. **35**(31), 2210878 (2023). <https://doi.org/10.1002/adma.202210878>
11. Y. He, J. Song, M. Li, K. Sakhatskyi, W. Li et al., Perovskite computed tomography imager and three-dimensional reconstruction. Nat. Photon. **18**(10), 1052–1058 (2024). <https://doi.org/10.1038/s41566-024-01506-y>
12. W. Qian, X. Xu, J. Wang, Y. Xu, J. Chen et al., An aerosol-liquid-solid process for the general synthesis of halide perovskite thick films for direct-conversion X-ray detectors. Matter **4**(3), 942–954 (2021). <https://doi.org/10.1016/j.matt.2021.01.020>
13. W. Pan, B. Yang, G. Niu, K.-H. Xue, X. Du et al., Hot-pressed CsPbBr_3_ quasi-monocrystalline film for sensitive direct X-ray detection. Adv. Mater. **31**(44), 1904405 (2019). <https://doi.org/10.1002/adma.201904405>
14. Y. Shen, Y. Liu, H. Ye, Y. Zheng, Q. Wei et al., Centimeter-sized single crystal of two-dimensional halide perovskites incorporating straight-chain symmetric diammonium ion for X-ray detection. Angew. Chem. Int. Ed. **59**(35), 14896–14902 (2020). <https://doi.org/10.1002/anie.202004160>
15. R. Zhuang, X. Wang, W. Ma, Y. Wu, X. Chen et al., Highly sensitive X-ray detector made of layered perovskite-like (NH_4_)_3_Bi_2_I_9_ single crystal with anisotropic response. Nat. Photonics **13**(9), 602–608 (2019). <https://doi.org/10.1038/s41566-019-0466-7>
16. Y. Liu, Z. Xu, Z. Yang, Y. Zhang, J. Cui et al., Inch-size 0D-structured lead-free perovskite single crystals for highly sensitive stable X-ray imaging. Matter **3**(1), 180–196 (2020). <https://doi.org/10.1016/j.matt.2020.04.017>
17. X. Zhang, D. Chu, B. Jia, Z. Zhao, J. Pi et al., Heterointerface design of perovskite single crystals for high-performance X-ray imaging. Adv. Mater. **36**(3), 2305513 (2024). <https://doi.org/10.1002/adma.202305513>
18. X. Xu, W. Qian, J. Wang, J. Yang, J. Chen et al., Sequential growth of 2D/3D double-layer perovskite films with superior X-ray detection performance. Adv. Sci. **8**(21), 2102730 (2021). <https://doi.org/10.1002/advs.202102730>
